# Regulating surface wrinkles using light

**DOI:** 10.1093/nsr/nwaa052

**Published:** 2020-03-28

**Authors:** Liangwei Zhou, Kaiming Hu, Wenming Zhang, Guang Meng, Jie Yin, Xuesong Jiang

**Affiliations:** School of Chemistry & Chemical Engineering, State Key Laboratory for Metal Matrix Composite Materials, Shanghai Jiao Tong University, Shanghai 200240, China; State Key Laboratory of Mechanical Systems and Vibration, School of Mechanical Engineering, Shanghai Jiao Tong University, Shanghai 200240, China; State Key Laboratory of Mechanical Systems and Vibration, School of Mechanical Engineering, Shanghai Jiao Tong University, Shanghai 200240, China; State Key Laboratory of Mechanical Systems and Vibration, School of Mechanical Engineering, Shanghai Jiao Tong University, Shanghai 200240, China; School of Chemistry & Chemical Engineering, State Key Laboratory for Metal Matrix Composite Materials, Shanghai Jiao Tong University, Shanghai 200240, China; School of Chemistry & Chemical Engineering, State Key Laboratory for Metal Matrix Composite Materials, Shanghai Jiao Tong University, Shanghai 200240, China

**Keywords:** surface patterns, ordered wrinkles, reversible buckling, photodimerization, dynamic light diffraction

## Abstract

Regulating existing micro and nano wrinkle structures into desired configurations is urgently necessary yet remains challenging, especially modulating wrinkle direction and location on demand. In this work, we propose a novel light-controlled strategy for surface wrinkles, which can dynamically and precisely regulate all basic characteristics of wrinkles, including wavelength, amplitude, direction and location (*λ*, *A*, *θ* and *L_c_*), and arbitrarily tune wrinkle topographies in two dimensions (2D). By considering the bidirectional Poisson's effect and soft boundary conditions, a modified theoretical model depicting the relation between stress distributions and the basic characteristics was developed to reveal the mechanical mechanism of the regulation strategy. Furthermore, the resulting 2D ordered wrinkles can be used as a dynamic optical grating and a smart template to reversibly regulate the morphology of various functional materials. This study will pave the way for wrinkle regulation and guide fabrication technology for functional wrinkled surfaces.

## Teaser

Wrinkles are widespread in nature and engineering, but hard to tune on demand. This article reports a light regulation strategy to realize ordered wrinkles with arbitrary configuration for various optical, electronic and sensor applications.

## INTRODUCTION

As the most ubiquitous surface pattern in both nature and engineering, wrinkles have attracted intense research interest for various applications, including flexible electronics [1–4], microfabrication [5,6], energy storage [7,8], microfluidic devices [9], smart displays [10,11] and anti-counterfeiting [12,13]. Regulating existing wrinkles into desired structures could deepen the understanding of wrinkling in nature and promote a number of interesting applications. For example, the dynamic switch of wrinkle amplitude (*A*) can help arteries adapt to different blood pressures and become self-cleaning to prevent formation of the nidus of a blood clot [14]. The specific distributions of the orientation (*θ*) and location (*L_c_*) of ridges on leaves and insect wings can transport necessary water and nutrients, offer mechanical support and even determine the shape of growth [15]. However, it remains a significant experimental and theoretical challenge to achieve ordered wrinkles by controlling their wavelength (*λ*), *A*, *θ* and *L_c_*.

Considerable regulation techniques of buckling surface instability, such as via a prepattern template, prestretching-release [16–19], mechanical compression [20,21], stretch-induced compression [22–24], solvent swelling [25,26], capillarity [27,28] and thermal expansion-compression [29–31], have been reported to tune wrinkle structures to some extent. Using a bas-relief-patterned substrate as a template, a locally ordered pattern can be obtained via the physical confinement effect [32–34], but this method is limited to multistep processes or the formation of a discrete and narrow strip layer. By using the external physical field, the above investigations tried to change some of the mechanical properties of layered systems to control wrinkle topographies, but the properties, especially the modulus and Poisson's ratio, are difficult to precisely control spatially and temporally. Therefore, these techniques lack the ability to precisely regulate a wrinkled system with arbitrary profiles, especially the reorganization of wrinkle orientation and location on a 2D surface.

In this paper, we propose a novel light-controlled strategy to arbitrarily regulate wrinkle topographies by precisely controlling the modulus spatial distributions and boundary conditions of the top layer for bilayer systems. This work, for the first time, can modulate all basic characteristics (*λ*, *A*, *θ* and *L_c_*) of wrinkles on demand, where previous studies could modulate only *λ* and *A* [29,35]. Moreover, by considering the bidirectional Poisson's effect and soft confinement effect, we also developed a modified shear-lag model to reveal the mechanical mechanism of the light-controlled strategy.

## RESULTS AND DISCUSSION

### Exposure strategy

**Figure 1.**
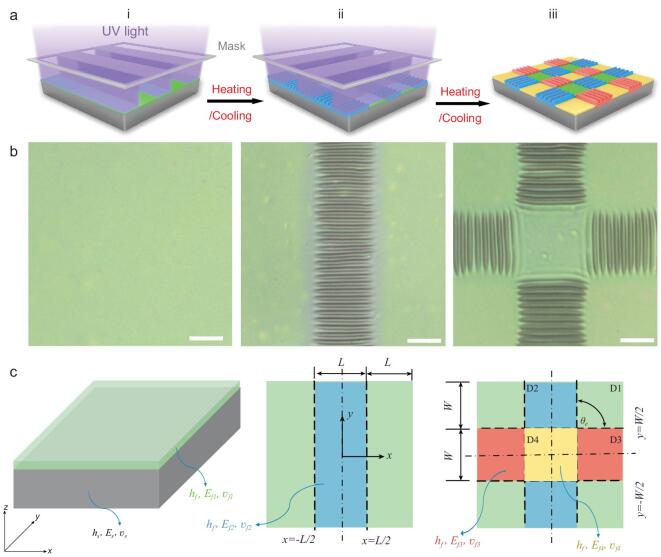
Fabrication, characterization and mechanical modeling of light-controlled wrinkle patterns by a lithography-compatible sequential exposure strategy. (a) A schematic diagram of the wrinkle fabrication process by sequential 365 nm UV light irradiation of a 200 μm strip array photomask. (b) The corresponding micrographs of the top film. Scale bars: 100 μm. (c) The mechanical modeling of a repeatable unit including domains D1–D4 in the film/substrate bilayer system, in which the boundary conditions for D1–D4 are *x* = ±*L*/2 and *y* = ±*W*/2, and *θ_e_* denotes the included angle of the photomask between two arbitrary exposures.

**Figure 2.**
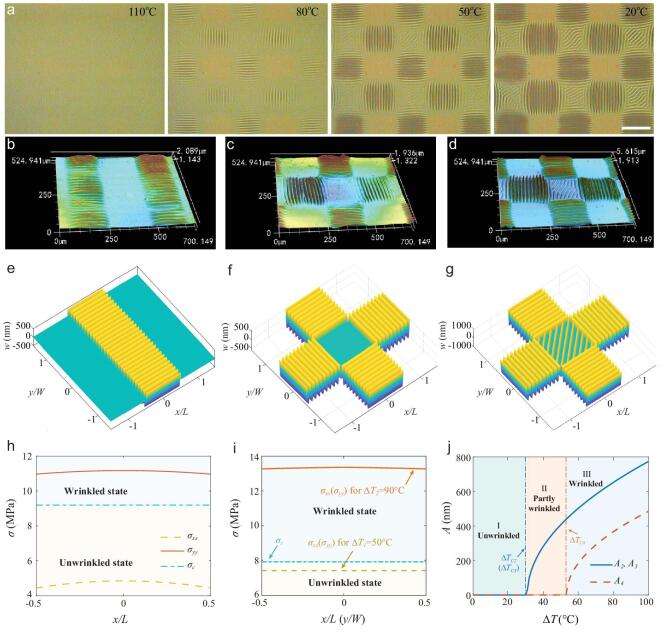
Surface wrinkle patterns induced by the sequential orthogonal exposure strategy at different temperatures. (a) Optical images of the wrinkle formation process after cessation of the heat treatment at 110°C. The room temperature was approximately *T_0_* = 20°C. Scale bar: 200 μm. (b, c) 3D LSCM images of strip wrinkle pattern and orthogonal wrinkle pattern (heating at *T_1_* = 70°C). (d) 3D LSCM image of orthogonal wrinkle pattern (heating at *T_2_* = 110°C). Images size = 525 × 700 μm. (e–g) The corresponding theoretical contours of the wrinkle patterns generated by the three different exposure strategies. (h) The stress distribution in the strip wrinkle pattern of the film after the first exposure. (i) The stress distributions of the film in D4 for two heating temperature differences $\Delta {T_{\mathit 1}} = {T_{\mathit 1}} - {T_{\mathit 0}}$ and $\Delta {T_{\mathit 2}} = {T_{\mathit 2}} - {T_{\mathit 0}}$. (j) The effect of the temperature on the amplitudes of D2, D3 and D4, where$\Delta {T_{C2}},{\rm{ }}\Delta {T_{C3}}$ and $\Delta {T_{C4}}$ are the critical temperature differences at which the wrinkles are triggered, respectively.

The unique advantages of light (e.g. noninvasive and neatness, as well as a high resolution for spatial and temporal control and a suitability for remote operation) in design of material surface characteristics suggest that light treatment may be a suitable way to regulate the local internal stresses in a wrinkled bilayer system with arbitrary profiles. We first fabricated and regulated 2D ordered wrinkles on bilayer systems by controlling the modulus of the top photosensitive layer spatially and temporally via a sequential exposure of UV light (Fig. 1). The bilayer system was prepared by spin-coating of an anthracene-contained copolymer (PAN-BA) on a substrate of poly(dimethylsiloxane) (PDMS) elastomer. PAN-BA was selected as the top layer because of its highly tunable photocrosslinking density and Young's modulus by the fast photodimerization of anthracene [36]. Additional details on synthesis and characterization of PAN-BA are provided in the Supplementary data (Figs S1–S3). Upon irradiation by 365 nm UV light for 15 min with a strip photomask and a further heating treatment at 70°C for 3 min, 1D ordered wrinkles were generated spontaneously in the exposed domain after cooling to room temperature. This formation occurred because of the mismatch in the moduli and thermal expansion coefficients of the stiff skin layer and the soft substrate, which was caused by the photocrosslinking of the PAN-BA top layer (Figs 1b and 2b). After rotating the photomask horizontally with a certain angle *θ_e_* (such as 90° in the schematic diagram of Fig. 1a ii), the 1D ordered wrinkled surface was then further exposed for 15 min to yield 2D ordered wrinkles (Figs 1b and 2c) through the heating/cooling treatment (70*°*C to 20*°*C). In the whole article, the ‘1D’ or ‘2D’ ordered wrinkles are used to describe the wrinkle patterns induced by a single or sequential exposure of UV light, respectively. And ‘1D’ or ‘2D’ ordered wrinkles are defined as wrinkles having one or multi-directions in a two-dimensional surface, respectively. The above sequential exposure strategy is lithography-compatible. As a result of sequential exposures when rotating the photomask, a repeatable unit of the top film/substrate system was divided into four rectangular domains with length *L* and width *W*, that is unexposed domain D1, first-exposure domain D2, second-exposure domain D3 and double-exposure domain D4 (Fig. 1c). It is noted that the yellow domains D4 represent two-step overlapping exposures that had been illuminated twice. The Young's moduli and Poisson's ratios of the surface films in D1, D2, D3 and D4 are *E*_*f1*_, *E*_*f2*_, *E*_*f3*_ and *E*_*f4*_, and *υ*_*f1*_, *υ*_*f2*_, *υ*_*f3*_ and *υ*_*f4*_. Because of the two equal orthogonal exposures, the Young's modulus distribution in the top film yields *E*_*f1*_*<E*_*f2*_ = *E*_*f3*_*<E*_*f4*_. What is surprising is that the resulting wrinkles do not form a continuous crisscross pattern in the exposed domains, as predicted, but rather a discrete and orthogonal highly ordered wrinkle pattern in confined domains. According to traditional buckling theory, because of the higher Young's modulus, the wrinkles in D4 should be generated with a larger amplitude than those in D2 and D3. In contrast, the wrinkles in D4 generated in the first exposure were then erased by the second irradiation.

To reveal the mechanical mechanism for the above intriguing phenomenon, a modified shear-lag model with bidirectional Poisson's effect is proposed to explain the evolution of the wrinkle patterns by the sequential exposure strategy (Fig. 1b). As seen in Fig. 1c, the photocrosslinked PAN-BA/PDMS bilayer can be considered as a stiff thin film with thickness *h*_*f*_ resting on a compliant soft substrate with thickness *h_s_*, Young's modulus *E_s_* and Poisson's ratio *v_s_*. The mechanical properties of the films, such as the Young's modulus, Poisson's ratio and mechanical boundary conditions, can be spatially reconfigured and then used to regulate the stress distributions and subsequent morphological evolution of the surface film. It has been demonstrated that Poisson's effects can greatly affect the wrinkle patterns of strip-like samples [37,38]. The strains in a strip-like sample, both along the ribbons and perpendicular to them, can be created by compressing or stretching the sample parallel to the long dimension of the ribbons [39]. Wang *et al.* [38] demonstrated that a large Poisson's effect can be introduced into a network-elastomer bilayer to suppress surface instabilities. In our work, as indicated in Fig. 1, the sequential exposure strategy can form two strip-like exposure domains. Therefore, there are two directions of Poisson's effects in the double-exposure domain D4. The spatial thermal stress reconfigurations of the surface film before stress relaxation are analysed by considering the bidirectional Poisson's effects in Fig. 1. The bidirectional Poisson's effects indicate that an additional strain $-{\nu_f}{\varepsilon _x}$ along the *y* direction can be induced by the thermal stress in the *x* direction and the additional strain $ - {\nu _f}{\varepsilon _y}$ along the *x* direction can also be induced by the thermal stress in the *y* direction (Fig. S4). When considering bidirectional Poisson's effects, the corresponding thermal stresses of D4 along the *x* and *y* directions can be expressed as

(1a) \begin{equation*} {\sigma _{x0}} = {\sigma _x} - {\nu _f}{\sigma _y}, \end{equation*}

(1b) \begin{equation*}{\sigma _{y0}} = {\sigma _y} - {\nu _f}{\sigma _x}.\end{equation*}

Then, the thermal-induced residual stresses can relax along both *x* and *y* directions because of the soft boundary conditions at *x* = ±*L*/2 or *y* = ±*W*/2 (Fig. S5). The modified shear-lag model is proposed to depict the 2D stress relaxation behavior of the sequentially exposed film/substrate system with a soft constraint effect, as detailed in Section S2.2 in Supplementary data. According to Eq. (S17), the stress distributions of the film/substrate system in D4 can be rewritten as

(2) \begin{equation*}\left[{\begin{array}{@{}*{1}{c}@{}} {{\sigma _{xx}}}\\ {{\sigma _{yy}}}\\ {{\sigma _{xy}}} \end{array}} \right] = \left[ {\begin{array}{@{}*{1}{c}@{}} {{f_1}\left( {\Delta T,{\upsilon _f},t,{\theta _e}} \right)}\\ {{f_2}\left( {\Delta T,{\upsilon _f},t,{\theta _e}} \right)}\\ {{f_3}\left( {\Delta T,{\upsilon _f},t,{\theta _e}} \right)} \end{array}} \right],\end{equation*}

where ${f_1},{\rm{ }}{f_2}$ and ${f_3}$ are given in Section S2.2 in Supplementary data. Furthermore, $\Delta T$ is the temperature difference of the heat treatment, which can be used to control the initial thermal stress ${\sigma _0}$ via the initial nonlocal physical field; ${\upsilon _f}$, *t* and ${\theta _e}$ are Poisson's ratio, the exposure time and the exposure included angle, respectively, which can be used to control the local stress field via the spatial mechanical properties of layered systems. As indicated by Eq. (2), the wrinkles in the film/substrate system can not only be tuned through a nonlocal regulation strategy but also modulated via a local strategy.

The intrinsic mechanical mechanism for the above experimental phenomena is revealed by the theoretical model of Eq. (S12), which is obtained from Eq. (2) by setting ${\theta _e} = {90^ \circ }$. For the film under a single exposure (Fig. 1a ii and Fig. 2b), the initial thermal stress can relax along the *x* direction at the soft boundary (*x* = ±*L*/2); therefore, ${\sigma _{xx}} < {\sigma _c} < {\sigma _{yy}}$ (Fig. 2h and Fig. S11a), and a 1D ordered wrinkle pattern can form along the *y* direction both experimentally and theoretically (Fig. 2b, e). However, the 1D ordered wrinkle pattern in D4 is erased after the second exposure. Generally, for the film under an additional exposure with ${\theta _e} = {90^ \circ }$ and *L* = *W* (Fig. 1a iii and Fig. 2c), the critical compressive stress of buckling in D4 (${\sigma _{c4}} =\! 7.91{\rm{ MPa}}$) is smaller than those in D2 and D3 (${\sigma _{c2}}\,\,{\rm{ and }}\,\,{\sigma _{c3}} = 9.04\,\,{\rm{ MPa}}$) because *E*_*f4*_*> E*_*f2*_ (*E*_*f3*_) (Fig. 2h, i and Fig. S11b). Based on the classical buckling model [32], the film in D4 can buckle into wrinkles before the single-exposure domains because of the elastic modulus enhancement induced by the sequential exposure. Therefore, the theoretical results under the classical model are not consistent with the experimental results in Figs 1b and 2c.

The reason for this is that the stress in D4 can be altered locally through Poisson's effect along both *x* and *y* axes although the initial nonlocal thermal stress ${\sigma _0}$ is fixed (Fig. S4). According to Eq. (2) and Fig. 2i, the thermal stress with consideration of the bidirectional Poisson's effect reduces to ${\sigma _0}( {1 - {\upsilon _f}} )$, which is a consequence of diminishing the stresses ${\sigma _{xx}}$ and ${\sigma _{yy}}$ in D4. As shown in Fig. 2i, both ${\sigma _{xx}}$ and ${\sigma _{yy}}$ range from 7.31 MPa to 7.43 MPa when *x* increases from ±*L*/2 to 0 or *y* increases from ±*W*/2 to 0. As a result of the bidirectional Poisson's effect, the residual thermal stresses ${\sigma _{xx}}$ and ${\sigma _{yy}}$ are smaller than the critical stress ${\sigma _{c4}}\,\,( { =\! 7.91{\rm{ MPa}}} )$ for $\Delta {T_1} = {50^ \circ }{\rm{C}}$, namely, ${\sigma _{xx}} = {\sigma _{yy}} < {\sigma _{c4}}$ (Fig. 2i), which suggests that neither of the residual thermal stresses (${\sigma _{xx}}$ and ${\sigma _{yy}}$) can trigger the buckling instability to form the wrinkles in D4 (Fig. 2i). Although the elastic modulus enhancement caused by the sequential exposure can lower the critical stress in D4, the bidirectional Poisson's effect can also reduce the residual stresses ${\sigma _{xx}}$ and ${\sigma _{yy}}$. Therefore, for the sample thermally treated at a low temperature, the bidirectional Poisson's effect leading to decrease in stress will dominate over the elastic modulus enhancement induced by the sequential exposure. According to the stress analysis, we attribute the above wrinkle pattern evolution in the double-exposure domain to the competitive mechanism between the stress relaxation caused by the bidirectional Poisson's effect and the elastic modulus enhancement induced by the sequential exposure (additional details are provided in Section S2.6 in Supplementary data).

As theoretically predicted in Eq. (2), when the temperature of the thermal treatment is high enough to induce compressive stress higher than the critical value, the buckling instability will also be theoretically triggered in the double-exposure domain D4 with the higher Young's modulus. Therefore, we heated the sample in Fig. 1a iii at a higher temperature (110°C) for 3 min and traced the wrinkle evolution by cooling the sample to room temperature (Fig. 2a). We saw that the wrinkles were first generated in domains D2 and D3 at 80°C and then appeared in domain D4 at 50°C during the cooling process (Fig. 2d). This experimental phenomenon is consistent with the prediction of our theoretical model that a larger temperature difference can result in a larger compressive stress, which then triggers the new wrinkle generation in D4 (Fig. 2i). For the temperature difference $\Delta {T_2} = {90^ \circ }{\rm{C}}$, the theoretical results in Fig. 2i indicate that both ${\sigma _{xx}}$ and ${\sigma _{yy}}$ range from 13.16 MPa to 13.37 MPa when *x* increases from ±*L*/2 to 0 or when *y* increases from ±*W*/2 to 0. In light of Eq. (S1), the initial external thermal stress ${\sigma _0}$ increases with the temperature, which increases the corresponding stresses beyond the critical stress ${\sigma _c}( { =\! 7.91{\rm{ MPa}}} )$, namely, ${\sigma _c} < {\sigma _{xx}} = {\sigma _{yy}}$. Therefore, the surface film in D4 buckles along both *x* and *y* axes when $\Delta {T_2} = {90^ \circ }{\rm{C}}$ (Fig. 2d). In this case, although the stresses in the surface film can be reduced by the bidirectional Poisson's effect, the residual thermal stresses are larger than the critical stress, and the thermal stress enhancement effect caused by the external physical field dominates over the bidirectional Poisson's effect.

Figure 2e–g shows the theoretical contours of wrinkle patterns generated under three different exposure strategies (calculated using the theoretical formula in Section S2.5 in Supplementary data), which are in good agreement with the experimental observation results (Fig. 2b–d). The wrinkle pattern evolution with increasing temperature can be elucidated by the theoretical curves, as shown in Fig. 2j. The critical temperature differences $\Delta {T_{C2}}$ and $\Delta {T_{C3}}$ of D2 and D3 are smaller than $\Delta {T_{C4}}$ of D4. As the temperature increases, there are three different phases of the wrinkle pattern. For phase I, $\Delta T < \Delta {T_{C2}}( {\Delta {T_{C3}}} ) < \Delta {T_{C4}}$, which indicates that the buckling instability cannot be triggered in these three domains D2, D3 and D4; for phase II, $\Delta {T_{C2}}( {\Delta {T_{C3}}} ) < \Delta T < \Delta {T_{C4}}$, which indicates that the wrinkle patterns in D2 and D3 can be generated, while the buckling instability cannot be triggered in D4; and for phase III, $\Delta {T_{C2}}( {\Delta {T_{C3}}} ) < \Delta {T_{C4}} < \Delta T$, which reveals that the wrinkle patterns can form in both single- and double-exposure domains. The dynamic evolution of the wrinkle pattern as the sample was cooled from 110°C (Fig. 2a) agrees with the theoretical results in Fig. 2j, which further validates the rationality of our modified theoretical model with the bidirectional Poisson's effect. The above results and discussions demonstrate that the wrinkle pattern evolution can be attributed to the competitive mechanism between the bidirectional Poisson's effect, the elastic modulus enhancement effect induced by multiple exposures and the thermal stress enhancement effect controlled by the external physical field (further details on the competitive mechanism can be seen in Section S2.6 in Supplementary data). The above competitive mechanism can be used to develop novel regulation methods for the wrinkle morphology.

Furthermore, the wrinkle morphology was also traced and checked by light diffraction to verify the validity of our theoretical model. The optically transparent wrinkle samples, which were transmitted by a green laser light (*λ* = 532 nm), projected Bragg diffraction images with distinct 3rd or 4th order spots onto a black screen (Figs S9 and S10). This result is consistent with the 3D laser scanning confocal microscope (LSCM) images in Fig. 2b–d, revealing that the larger amplitude of wrinkles allows a higher diffraction efficiency. For the temperature difference $\Delta {T_2} = {90^ \circ }C$, the crossed diffraction image transformed into a coexisting cross and ring image (Fig. S10b and c). This result is in accord with the theoretical model that proves how the new wrinkles were generated in domain D4. Additionally, the orientation of the discrete diffraction spots is parallel to the arrangement direction of the sinusoidal wrinkle waves with the decreasing intensity from the center to the edge (Fig. S10). The high quality of the light diffraction images also suggests that the wrinkles are highly 2D ordered over the whole surface.

### Regulating 2D ordered wrinkle morphology by selective UV irradiation

**Figure 3.**
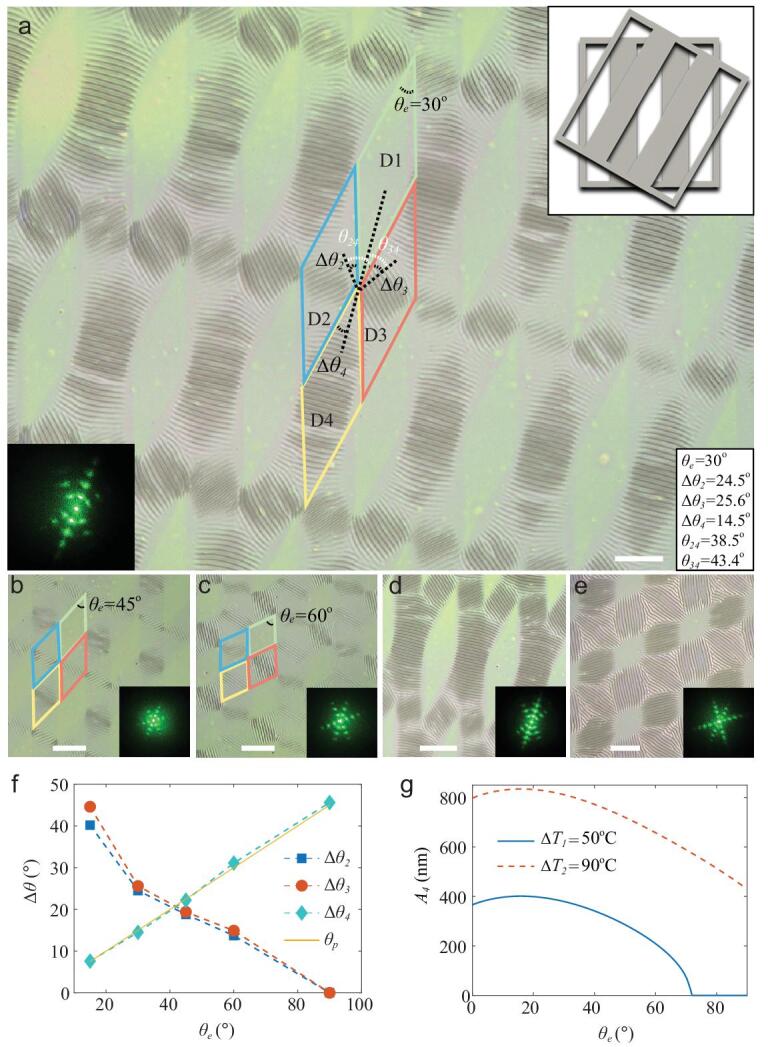
Regulation of 2D ordered wrinkle morphologies under various exposure included angles. (a–c) The 2D micrographs of the wrinkles fabricated with the included angles *θ_e_ =* 30°, 45°, 60° (thermal treatment of *ΔT_1_ =* 50°C). The insets reveal the second laying directions of photomask and the corresponding diffraction patterns. Scale bars: 200 μm. (d, e) The 2D micrographs of the corresponding wrinkle samples (*θ_e_ =* 30° and 60°) were further heated at a higher temperature difference of *ΔT_2_ =* 90°C. The insets represent the corresponding diffraction patterns. Scale bars: 200 μm. (f) The experimental orientation rotations *Δθ_2_* and *Δθ_3_* of the wrinkles in D2 and D3 with respect to different *θ_e_* values, and a comparison of the experimental *Δθ_4_* in D4 and the theoretical principal angle *θ_p_* with respect to *θ_e_*. (g) The effect of *θ_e_* on the amplitude of the wrinkle pattern in D4 for two different temperatures *T_1_* and *T_2_*.

As the orientation of compressive stress determines the arrangement direction of the wrinkles, we regulated the formation and morphology of different wrinkle domains through a sequential selective exposure strategy, such as controlling exposure including angle *θ_e_* and exposure time. In the selective UV irradiation strategy, the samples were first placed under a strip photomask for 15 min and then irradiated under the same photomask arranged in different directions for the same time (Fig. 3a–c and Fig. S12). The wrinkle patterns in the 2D plane possess three defined directions, which change with *θ_e_* adjusted from 30° to 60°, and consequently, the amplitude of wrinkles in D4 decreases significantly. The diffraction spots also exhibit three directions, in keeping with the wrinkle orientations. *θ_ij_* is defined as the included angle between the wrinkle orientations in Di and Dj. As shown in Fig. S13a, when *θ_e_* = 30°, *θ_24_* and *θ_34_* are 38.5° and 43.4°, respectively, and the included angles of the diffraction spots are *θ_d_* = 38.3° and 42.9°, respectively (Fig. S13b). When *θ_e_* = 45° and 60°, similar results are seen (Fig. S13c–f). These experimental results are almost consistent with the theoretical results of the geometric angle relationship: *θ_24_* = *θ_34_* = *θ_d_*. Therefore, when the exposure time is fixed, the first and second exposures are equivalent to regulate the wrinkles. In addition, the wrinkle morphology can be modulated again by heat treatment. When the samples were further heated at 110°C for 3 min, all the exposure domains generated evident wrinkles accompanied by a larger diffraction order (Fig. 3d, e and Fig. S14).

According to the theoretical stress analysis for the film/substrate system by Eq. (S16), the directions of the wrinkles dominated by the value and direction of the principal stresses can be regulated by angle *θ_e_* of the photomask. Figure 3 and Fig. S13 present the experimental tuning results of *θ_e_* on the orientations of the wrinkle pattern. As seen in Fig. S13, when *θ_e_* = 30°, the orientation of the wrinkle pattern in D2 is rotated counterclockwise by *Δθ_2_* = 24.5°, and the orientation in D3 is rotated clockwise by *Δθ_3_* = 25.6°. When *θ_e_* increases to 45°, *Δθ_2_* = 18.8° and *Δθ_3_* = 19.4°. When *θ_e_* = 60*°*, the orientations in D2 and D3 are rotated only by 13.7° and 14.9°, respectively. These experimental results are consistent with the theoretical results of the geometric angle relationship: *Δθ_2_* = *Δθ_3_*. Furthermore, as shown in Fig. 2c, the orientations exhibit no rotation when *θ_e_ =* 90*°*.

Here, we demonstrate that the values of orientation rotations for the wrinkles (*Δθ_2_* and *Δθ_3_*) in D2 and D3 decrease when *θ_e_* increases (Fig. 3f). The mechanical mechanism for the orientation rotation of the wrinkle pattern is the soft constraint boundary. As shown in Fig. S6c and Eq. (S5), the stresses in D4 at the boundaries (*x* = ±*L* and *y* = ±*W*) ${( {{\sigma _{xx}}} )_b}$ and ${( {{\sigma _{yy}}} )_b}$ can supply the shear stresses ${( {{\sigma _{xy}}} )_b} = {( {{\sigma _{xx}}} )_b}\cos {\theta _e}$ and ${( {{\sigma _{xy}}} )_b} = {( {{\sigma _{yy}}} )_b}\cos {\theta _e}$. According to Eqs (S25) and (S26), the shear stress ${( {{\sigma _{xy}}} )_b}$ can change the principal stresses and the corresponding principal angle *θ_p_* of the single-exposure domain, which rotates the wrinkle orientation. Moreover, both *Δθ_2_* and *Δθ_3_* decrease with the increase of *θ_e_*. The reason is that the shear stress ${( {{\sigma _{xy}}} )_b}$ and principal angle *θ_p_* decrease with increasing *θ_e_*. However, as seen in Fig. 3f, the theoretical *Δθ_4_* in D4 increases with *θ_e_*. The experimental *Δθ_4_* in D4 is consistent with the theoretical principal angle *θ_p_* and can be given as: *Δθ_4_* = *Δθ_p_* = 1/2*θ_e_*. The intrinsic mechanical explanation is that *θ_p_* in D4 increases with *θ_e_*. Based on the above analysis, we obtain the geometric relationship between *θ_24_*, *Δθ_2_* and *Δθ_4_* as *θ_24_* = *Δθ_2_*+*Δθ_4_*.

Moreover, *θ_e_* can also impact the amplitude of the wrinkles in the double-exposure domain. As shown in Fig. 3, when *θ_e_* increases from 30° to 60°, the observed amplitude of the wrinkles in D4 decreases, which matches well with the theoretical results (Fig. 3g). According to Eqs (S16) and (S25), we can obtain the following relationship $A \propto \sqrt {{\sigma _{\mathit 1,2}}} \propto $$\sqrt {( {1 - {\upsilon _f}\sin {\theta _e}} )( {\sin {\theta _e} + \cos {\theta _e}} )} $, namely, the principal stresses ${\sigma _{{\mathit{1}},{\mathit{2}}}}$ and the theoretical amplitude *A_4_* of the wrinkles in D4 can first increase and then decrease as *θ_e_* increases from 0 to 90°.

**Figure 4.**
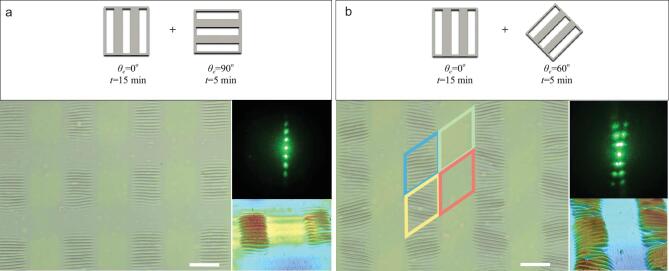
Regulation of wrinkles by sequential UV irradiation with unequal time. (a) A 2D micrograph of the strip wrinkles exposed to 15 min of 365 nm UV irradiation and a further 5 min of 365 nm UV irradiation with *θ_e_* = 90°. (b) A 2D micrograph of the strip wrinkles exposed to 15 min of 365 nm UV irradiation and a further 5 min of 365 nm UV irradiation with *θ_e_* = 60°. The insets are the corresponding diffraction patterns and 3D LSCM images of the wrinkles. Scale bars: 200 μm.

Generally, the wrinkle direction is difficult to be tuned on demand once the wrinkles form and are fixed, in spite of considerable efforts in the fabrication of desired ordered wrinkles [33,40,41]. The above experimental and theoretical results suggest that reconfiguring the wrinkle characteristics through sequential control of the spatial modulus of the PAN-BA layer is possible via post-UV irradiation. Thanks to the high spatial resolution and temporal controllability of light, we can regulate not only the amplitude and wavelength but also the orientation of the generated wrinkles, independently. Instead of fixing the same irradiation time in the sequential exposure process, the second exposure time was cut to 5 min, which is too short to trigger the wrinkle formation independently. As shown in Fig. 4a, when *θ_e_ =* 90°, the initial 1D ordered wrinkles in the double-exposure domain are partially erased because the thermal-induced residual stresses were reduced to below the critical stress by the bidirectional Poisson's effects. When *θ_e_ =* 60°, surprisingly, the initial 1D ordered wrinkles appear distorted into two different directions, which is further confirmed by the two directions in the diffraction pattern (Fig. 4b). This result might be explained by the second exposure dividing the original 1D ordered wrinkles into a single-exposure domain and a double-exposure domain, with the directions in both D2 and D4 being changed as a result of the soft constraint boundary effect. We believe that this strategy to harness existing wrinkle structures into arbitrary configurations is an important breakthrough and a more practical method than just fabrication of desirable wrinkles.

In addition, because the light diffraction properties are strictly correlated to the surface morphology of the wrinkles, we can easily characterize the surface topography by the diffraction patterns of the samples. When a green laser was moved back and forth over different domains with the ordered wrinkles, the diffraction pattern changed between 1D and 2D as the microstructure changed, accordingly (Fig. S15, Movie S1). Therefore, the characteristics (*λ*, *A*, *θ* and *Lc*) of the wrinkle surface can be recorded by the characteristics (diffraction shape, diffraction orders and light intensity) of the diffraction pattern. Furthermore, the wrinkle surface can be dynamically recorded by a green laser and a red laser simultaneously (Fig. S16, Movie S2). This approach may offer a noncontact and efficient method to detect 3D microstructures of material surfaces without complicated experimental equipment and procedures.

### Dynamic optical grating and functionalized wrinkled platform

**Figure 5.**
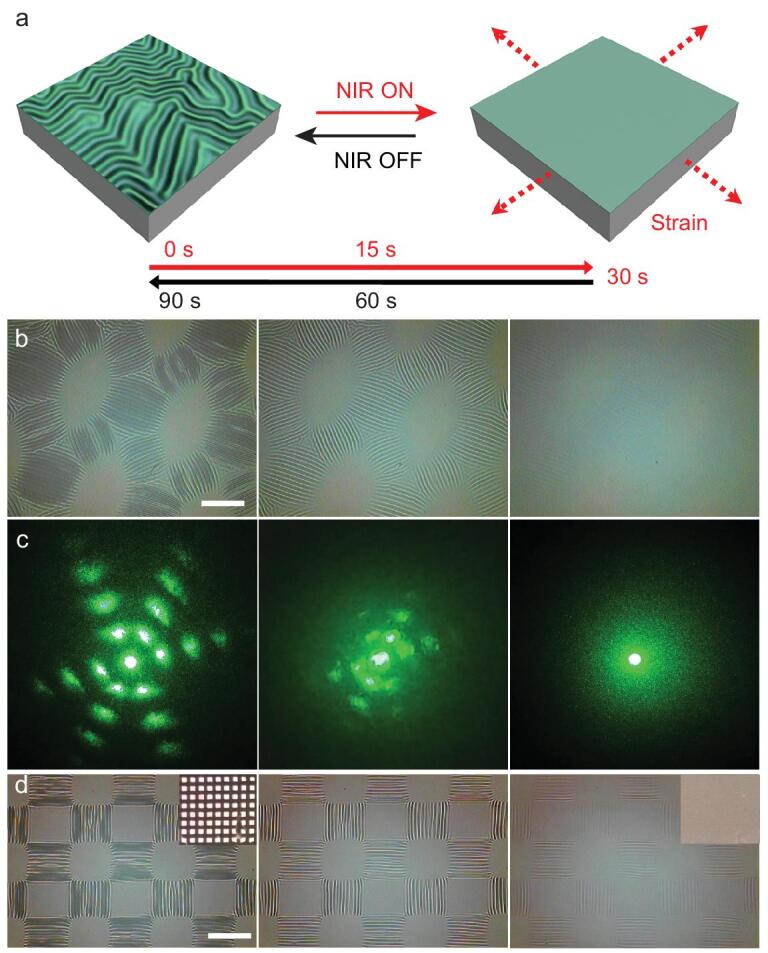
Illustration of the NIR-driven ordered wrinkle serving as a dynamic light grating and a functionalized wrinkled platform. (a) Schematic illustration of the NIR-driven dynamic wrinkling by controlling the strain via the photo-to-thermal energy conversion of NIR. (b) Optical images of the wrinkle disappearance/regeneration process by the NIR on/off switch. (c) The corresponding light diffraction patterns. (d) Optical images of Au-functionalized wrinkle disappearance/regeneration process by NIR on/off switch. The insets are the corresponding macrophotographs by camera. The NIR light intensity was 1.5 W/cm^2^. Scale bar: 200 μm.

In addition to the sequential UV irradiation to regulate the wrinkles by controlling the spatial modulus of the PAN-BA top layer, NIR can be further used to tune the resulting wrinkles by controlling the compressive strain in the bilayer system. We used PDMS-containing carbon nanotubes (CNT-PDMS, 0.05 wt%) as an elastomer substrate for the bilayer system [35]. The thermal expansion of the CNT-PDMS substrate can be reversibly regulated by 808 nm NIR irradiation, and the resulting wrinkles can be erased *in situ* and in real time through the high photon-to-thermal energy conversion efficiency (Fig. 5a). The NIR-driven hierarchical pattern can be reconfigured between the wrinkled state and the smooth state (Fig. 5b, Fig. S17, Movies S3, 4). Furthermore, the controllable NIR light erasure and regeneration switch for wrinkle patterns can be used in reverse to regulate light diffraction as a dynamic optical grating. Although various micro-/nanostructures can be prepared as static gratings, dynamic gratings remain a challenge because of difficulty in reversibly transforming the grating topography from a structured state to a flat state *in situ*. A few studies have reported realization of dynamic grating by liquid crystal materials [42,43], but these optical gratings may possess limitations in response speed, grating morphology and complicated fabrication processes. Fig. 5c and Movie S5 show the NIR-driven light grating for dynamic modulation of light diffraction. When a green laser passed through the 2D ordered wrinkled sample, a discrete 2D diffraction pattern was observed, and the two adjacent spots on the same axis were almost equidistant. When the wrinkled surface was irradiated by NIR, the 3rd diffraction pattern quickly disappeared within 30 s; the process initiated at the outside spots, progressed toward the inner spots via gradual wrinkle erasure and then became a central spot after the wrinkled surface was fully levelled (Fig. 5b and c). When the NIR light was turned off, the diffraction pattern was gradually restored to its initial state through regeneration of the wrinkles. Moreover, based on our ability to write arbitrary highly ordered surface wrinkle patterns, a series of 1D and 2D dynamic light gratings can be obtained and cycled more than 1000 times without any damage to the wrinkle morphology (Fig. S17). The controlled reversibility of dynamic wrinkle patterns can enable fabrication of novel optical devices and architectures, as well as greatly advance fundamental understanding of the temporal nature (such as wettability, friction and adhesion) of flexible materials influenced by external stimuli.

Furthermore, the bilayer system can be used as a dynamic template, which can deposit or transfer various functional molecules including conductor, semiconductor and insulator onto this bilayer to form a three-layer system: the upper layer, middle layer and substrate can act as a functional layer, a morphology control layer and a dynamic response layer, respectively (Fig. S18a). For example, we deposited 10 nm Au onto the bilayer, the wrinkle topography was still well regulated by UV light-responsive PAN-BA film and could be dynamically erased/restored by NIR light-responsive CNT-PDMS substrate (Fig. 5d and Fig. S18b). Similarly, the semiconductor Ge was also deposited on the bilayer to prove the universality and versatility of this strategy (Fig. S18c). In other words, by regulating the wrinkle characteristics including *λ*, *A*, *θ* and *L_c_* of the dynamic template, we can easily fabricate different periodic functionalized microstructures on demand. We believe that the three-layer or even multi-layer wrinkled platform enables various functional layers being ordered and being dynamic transformed on 2D surface. This powerful functionalized wrinkled platform may be used for flexible electronics, microfabrication, sensors and micro-/nanochannels.

## CONCLUSION

In summary, we demonstrated a sequential exposure strategy to spatially and temporally regulate the morphology (*λ*, *A*, *θ* and *L_c_*) of 2D ordered wrinkles on a bilayer system comprising photocrosslinked PAN-BA as the top layer and CNT-PDMS as the substrate. The key point to the regulation of 2D ordered wrinkles is that the mechanical properties of the bilayer system can be precisely controlled by photodimerization. We further developed a modified shear-lag model with a bidirectional Poisson's effect and soft confinement effect to understand the 2D spatial distribution of the mechanical property for the top layer, which can determine the stress field and wrinkle topography. The theoretical stress analysis demonstrates that the wrinkle pattern evolution can be attributed to the competitive mechanism between the elastic modulus enhancement effect induced by sequential exposure and the thermal stress enhancement effect controlled by the external physical field. This study provides a general guideline to fabricate and regulate 2D ordered wrinkles on a surface and also offers a dynamic template to reversibly regulate the morphology of various functional materials. We envision that this light-induced dynamic and reconfigurable wrinkle pattern can be used as a smart surface platform for optical devices, sensors, flexible electronics, information storage and anticounterfeiting.

## METHODS

### Materials

Sodium methanolate (CH_3_ONa), potassium iodide (KI), 9-anthracenemethanol (AN-OH) and 4-vinylbenzyl chloride (S-Cl) purchased from China National Pharmaceutical Group were used directly without further purification. n-butyl acrylate (BA) obtained from China National Pharmaceutical Group was washed with saturated sodium hydroxide solution to remove retarder and then dried with magnesium sulfate. Multiwalled carbon nanotubes (CNT) were purchased from J&K Scientific Ltd. and were directly used.

### Fabrication and regulation of wrinkle patterns

Elastomeric PDMS sheets were prepared using a silicone elastomer (Sylgard 184, Dow Corning). The silicone base and curing agents were mixed at a 10:1 mass ratio in a polystyrene Petri dish and stirred at room temperature. Then, the mixture was baked at 70°C for 4 h, and then, degassed for 0.5 h to yield a crosslinked PDMS elastomer substrate. CNT-PDMS sheets were prepared in the same way as PDMS sheets, except for premixing 0.05 wt% of multiwalled carbon nanotubes (CNT) into the silicone base.

A 6% toluene solution of PAN-BA was first spin-coated on a PDMS (or CNT-PDMS) substrate and then irradiated with 365 nm UV light under a strip mask for the desired time, resulting in the relatively stiff domains on the top layer film. The coated PDMS substrate was heated to the desired temperature for 3 min and cooled down to room temperature to generate the ordered wrinkle patterns in the exposure domains. The ordered wrinkle patterns could be regulated by a second 365 nm UV light irradiation process for a certain time and orientation of the photomask.

### Characterization

Static wrinkle patterns were recorded with a 3D laser scanning confocal microscope (VK-X1000, KEYENCE). The dynamic wrinkle pattern elimination/reappearance process was recorded with a profile measurement microscope (VF-7501, KEYENCE). The Young's modulus of the top film was measured by atomic force microscopy nanomechanical mapping (Dimension Icon & FastScan Bio, Bruker), where the oscillation frequency of the Z-piezo was 1.0 kHz and the peak force amplitude was set at 150 nm. The samples were scanned using Olympus microcantilevers with a spring constant of 3 N/m. The NIR light was produced by a laser diode controller (*λ* = 808 nm; LE-LS-808-1000TFCB, LEO Photonics). ^1^H NMR spectra were measured on a Varian Mercury Plus spectrometer (400 MHz) with deuterated chloroform (CDCl_3_) as the solvent and tetramethylsilane as an internal standard at room temperature.

## Supplementary Material

nwaa052_Supplemental_Files
