# Supplementary material for: Regulating surface wrinkles using light
Source: Natl Sci Rev. 2020 Mar 28;7(7):1247–57. doi: 10.1093/nsr/nwaa052 (PMC8288942; doi:10.1093/nsr/nwaa052)
Supplement: nwaa052_Supplemental_Files [file nwaa052_supplemental_files.zip › NSR_MS-2020-168-SI.docx]

Supplementary information

**Regulating surface wrinkles using light**

Liangwei Zhoua,1, Kaiming Hub,1, Wenming Zhangb,*, Guang Mengb, Jie Yina, Xuesong Jianga,*

aSchool of Chemistry & Chemical Engineering, State Key Laboratory for Metal Matrix Composite Materials, Shanghai Jiao Tong University, Shanghai 200240, P.R. China

bState Key Laboratory of Mechanical Systems and Vibration, School of Mechanical Engineering, Shanghai Jiao Tong University, Shanghai 200240, P.R. China

1L. Zhou and K. Hu contributed equally to this work.

E-mail: ponygle@sjtu.edu.cn (X. Jiang), wenmingz@sjtu.edu.cn (W. Zhang)

**1. Experimental Section**

**1.1. Synthesis of Anthracene-containing Styrene Monomer (S**-**AN)**

A 150 mL tetrahydrofuran solution of anthracene-9-methanol (AN-OH, 4.16 g, 20 mmol) was prepared and purged with highly pure N2 gas for 30 min to eliminate oxygen. To this stirred solution, a tetrahydrofuran (7 mL) solution of sodium methoxide (CH3ONa, 5.4 g, 0.1 mol) and potassium iodide (KI, 16.7 g, 0.1 mmol) was added as solids at ambient temperature. Then, the reaction system was added drop-wise with a tetrahydrofuran (10 mL) solution of 4-(chloromethyl) styrene (S-Cl, 3.06 g, 20 mmol) in 30 min. Then, the reaction system was heated to 50 °C. After reacting for 24 h, the organic phase was collected, then precipitated in methyl alcohol. Then the yellow power precursor St-AN was obtained in quantitative yield by filtration and dried at 50 °C for 12 h. 1H NMR (500 MHz, CDCl3): 4.73 ppm (s, 2H), 5.29 ppm (d, 1H), 5.52 ppm (s, 2H), 5.81 ppm (d, 1H), 6.77 ppm (q, 1H), 7.39-7.57 ppm (m, 8H), 8.04 ppm (d, 2H), 8.34 ppm (d, 2H), 8.50 ppm (s, 1H).

**1.2. Synthesis of** **Poly (n-butylacrylate-co-anthracene-containing styrene) (PAN-BA)**

The procedure to synthesize the Poly (n-butylacrylate-co-anthracene-containing styrene) (PAN-BA) is shown in Fig. S1. A set amount of monomers BA and S-AN at the feed mole ratio was dissolved in 10 mL of pre-dried 1, 4-dioxane. The total mass of monomers mixture is 4 g. Then 40 mg (1 wt % of the total monomer weight) of 2,2-azobisisobutyronitrile (AIBN) was added, and the polymerization reactions were run at 70 °C for 24 h under nitrogen atmosphere. After cooling at room temperature, thereaction mixture was precipitated for three times in 100 mL of cold hexane. Then the copolymer was obtained in the quantitative yield by filtration and dried at 60 °C for 24 h. The structure of final product was verified by 1H NMR spectrum. The constituent ratio of the polymer is calculated by integrals in 1H NMR spectrum. The peak between above 6.0 was the signal of the proton connecting to anthracene and styrene. The signal below 1.0 belonged to the proton connecting to methyl of butyl-acrylate. And the result of actual constituent ratio in final copolymers was 1:5.5.


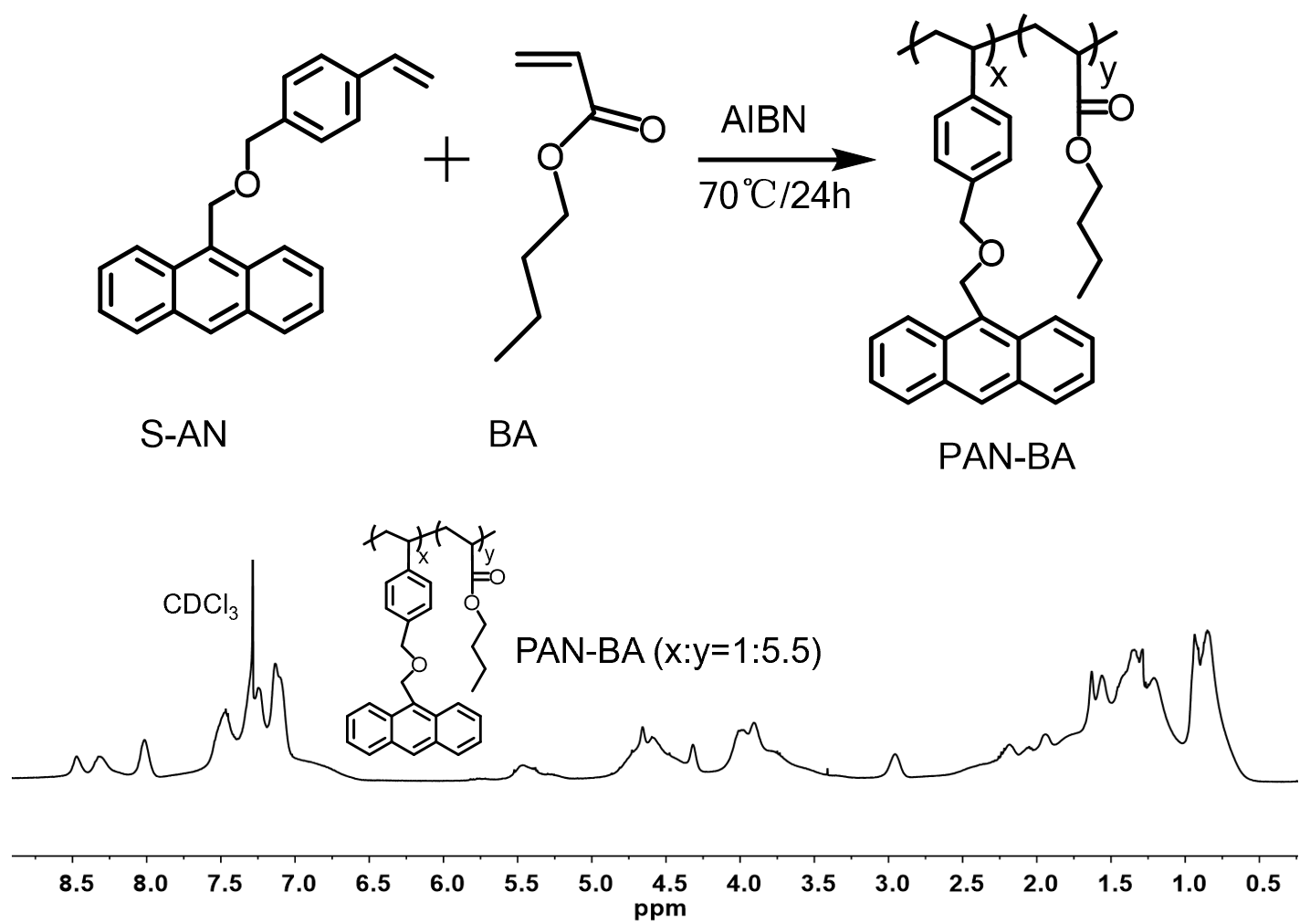


**Figure S1.** The synthesis and 1H NMR spectrum of PAN-BA in CDCl3.


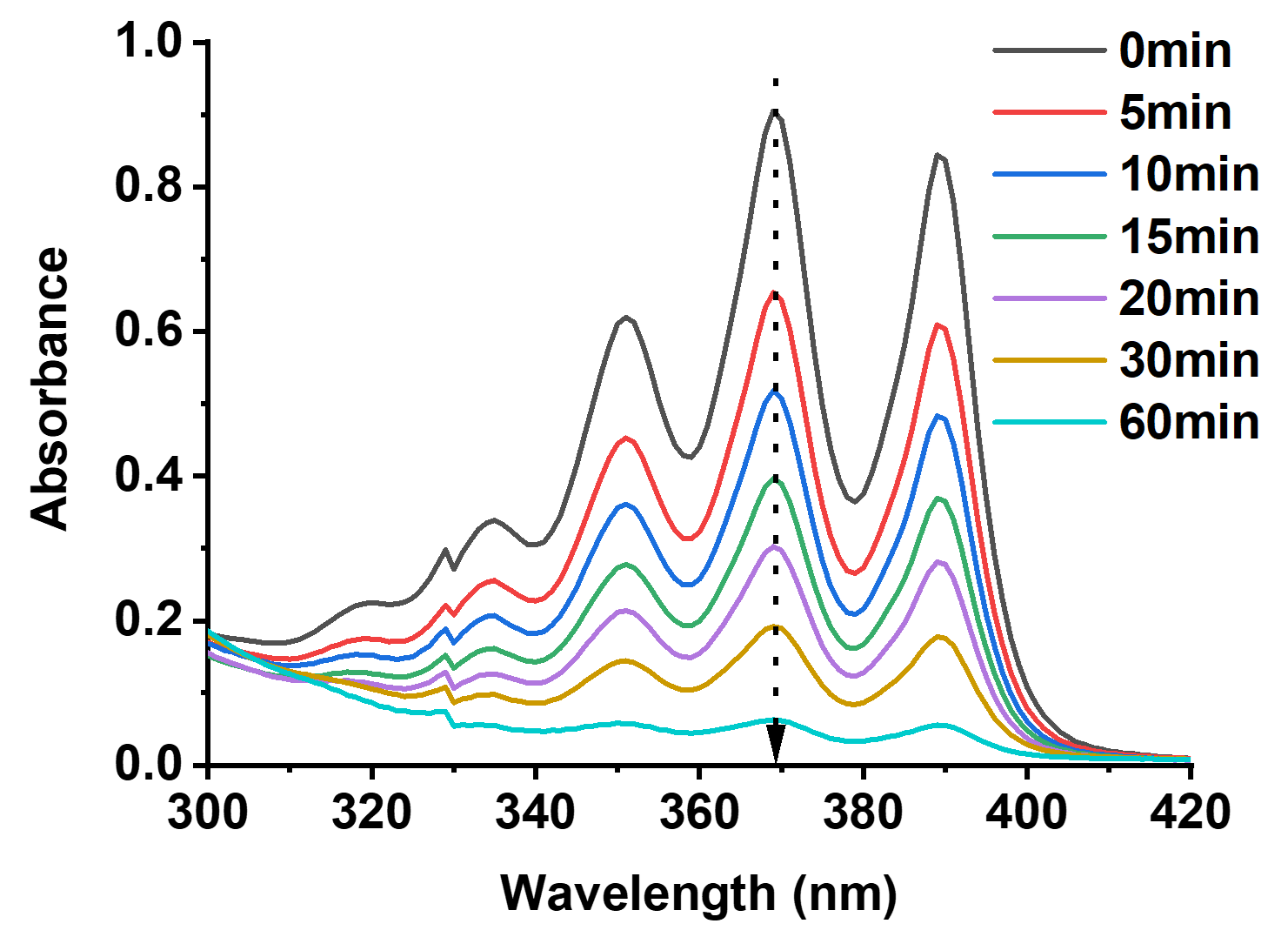


**Figure S2.** UV-Vis spectra evolution on photodimerization reaction of PAN-BA upon 365 nm UV exposure.

**Figure S3.** Young’s modulus Ef of the top film as a function of 365 nm UV light irradiation time

**2. Theoretical model**

**2.1 Bidirectional Poisson's effect**

The equi-biaxial compressive stresses induced by different thermal expansion coefficients between the surface thin film and PDMS substrates can be given by [1]

(S1)

where *Ef*denotes the Young’s modulus of the film, and are the thermal expansion coefficients of soft substrate and stiff thin film, respectively. denotes the temperature difference of the heat treatment. is the Poisson’s ratio of the surface film.

As shown in Fig. S4，thermal stress reconfigurations of the surface film before stress relaxation are analyzed by considering the bidirectional Poisson's effects. The bidirectional Poisson's effects indicate that the additional strain along the *y* direction can be induced by the thermal stress in the *x* direction (Fig. S4a) and the additional strain along the *x* direction can be also induced by the thermal stress in the *y* direction (Fig. S4b). The thermal strain in the double-exposure domains along *x* and *y* directions with considering bidirectional Poisson's effects can be obtained as

(S2a)

(S2b)

The corresponding thermal stresses of the double-exposure domains with considering bidirectional Poisson's effects can be expressed as

(S3a)

(S3b)


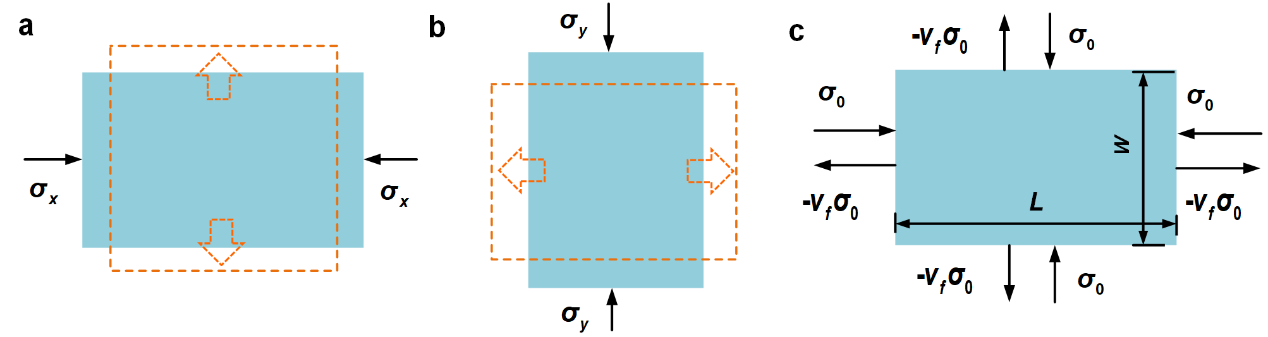


**Figure S4.** Bidirectional Poisson's effects of stress reconfigurations for the double-exposure domains with included angle of 90°. (a) The additional strain along the *y* direction can be induced by the thermal stress in the *x* direction. (b) The additional strain along the *x* direction can be also induced by the thermal stress in the *y* direction. (c) The additional strains alongthe *x* direction and the additional strain along the *y* direction under bidirectional Poisson's effects.

**2.2 Stress analysis of the double**-**exposure domains**

Then, thermal-induced residual stresses can relax along both *x* and *y* directions due to soft boundary conditions at *x*=±*L*/2 or *y*=±*W*/2. As shown in Fig. S5, the boundary of the double-exposure domains is single-exposure domains. Therefore, an equivalent stiffness is introduced to depict the stress relaxation in the double-exposure domains as follows

(S4)

where is the elastic modulus of the surface film in the single-exposure domains.

Judge from the experimental configurations, the soft boundary conditions of the double-exposure domains can be given by

(S5a)

(S5b)

where is the in-plane displacement of the neutral plane.


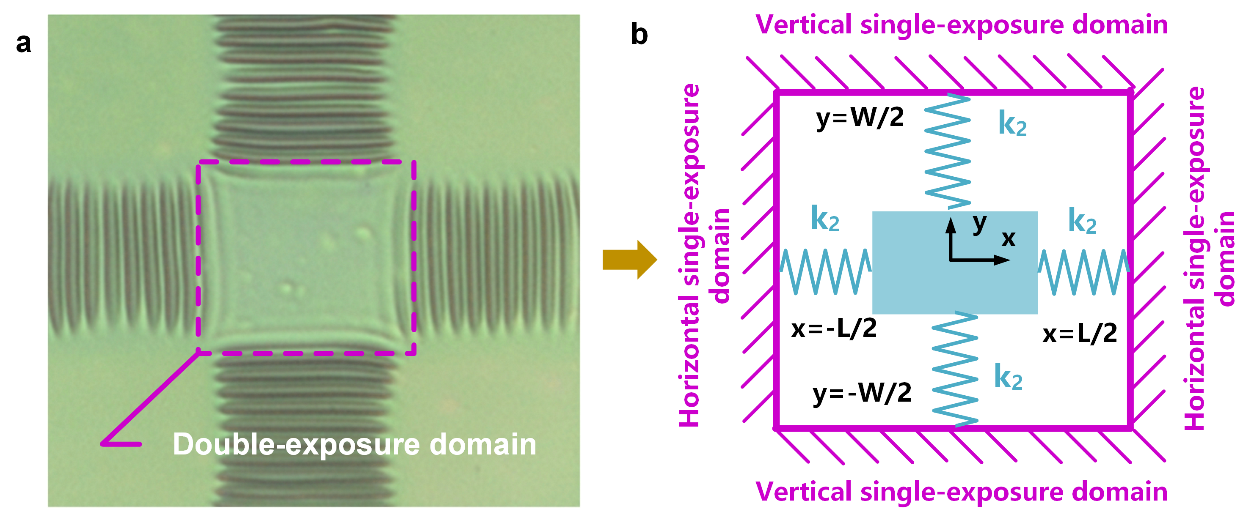


**Figure S5.** Mechanical modelling of soft boundary conditions for the double-exposure domains. (a) The experimental micro image of the double-exposure domains. (b) Equivalent mechanical boundaries of the double-exposure domains, where *k*2=*k*3.

In order to calculate the stress distribution of the double-exposure domains, the thermal-induced residual stresses can relax along the *x* direction. The stress in the *x* direction can be expressed as

(S6)

where the shear traction at the interface between the film and the substrate , denotes the elastic shear modulus of substrate at the rubbery limit.

According to the constitutive relation of the top film, the stresses in the thin film can be given by

(S7a)

(S7b)

Eq. (S7) can be solved with the boundary conditions Eq. (S5a) and Eq. (S6), the stress distributions of the surface film in the double-exposure domains after *x*-directional stress relaxation can be obtained as

(S8a)

(S8b)

where the term of ‘’ is induced by one-direction Poisson’s effect, the term of ‘’ is induced by soft boundary conditions, and the shear-lag length .

The thermal-induced residual stresses can also relax along the *y* direction at the soft boundary *y*=±*W*/2 and the stress can be expressed as

(S9)

where the shear traction at the interface between the film and the substrate , denotes the elastic shear modulus of the substrate at the rubbery limit.

According to the constitutive relation of the top film, the stresses in the thin film can be given by

(S10a)

(S10b)

Eq. (S10) can be solved with the boundary conditions Eqs. (S5b) and (S9), the stress distributions of the surface film in the double-exposure domains after *y*-directional stress relaxation can be obtained as

(S11a)

(S11b)

The stress distributions of the double-exposure domains after 2D stress relaxation can be approximately calculated by overlying the stress relaxation along both *x* and *y* directions due to the 2D shear-lag model is difficult to solve. Therefore, the 2D stress distributions after 2D stress relaxation can be expressed as

(S12a)

(S12b)

To obtain the stress distributions of the double-exposure domains when the included angle *θe* of a mask between two different exposures is arbitrary, the rotational transformation matrix of the coordinates is introduced as shown in Fig. S6b. The transformation matrix can be expressed as

(S13)

**
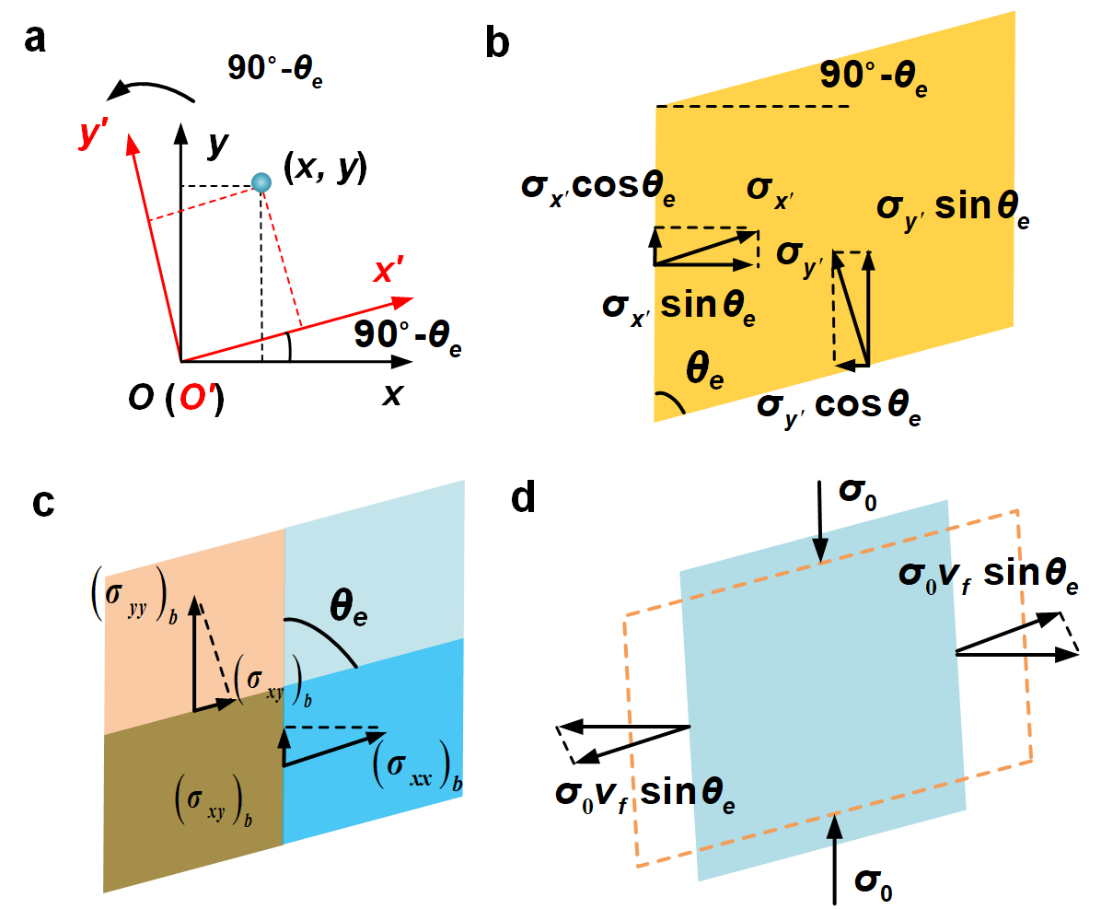
**

**Figure S6****.** Stress analysis of the double-exposure domains with the included angle *θe*.(a) Rotational transformation matrix of coordinates. (b) Stress distributions of the double-exposure domains. (c, d) Stress analysis at boundaries.

Combine Eq. (S12) with Eq. (S13), we obtain the stress distributions of the double-exposure domains with arbitrary *θe*as follows

(S14a)

(S14b)

The stresses in the double-exposure domains with an included angle *θe* can be expressed in *x-y* coordinate system as follows

(S15a)

(S15b)

(S15c)

Substituting Eq. (S14) into Eq. (S15) yields

(S16a)

(S16b)

(S16c)

Because initial thermal stress , and are determined by initial elastic modulus *Ef1* and the exposure time *t*, Eq. (S16) can be rewritten as follows

(S17)

where

Eq. (S12) can also be obtained by Eq. (S16) when. In order to calculate the value of stress in surface film, the values of the following parameters are given as follows; , ; ; .

**2.3 Stress analysis of the single**-**exposure domains**

For the single-exposure domains, the boundaries of the single-exposure domains are unexposure domains. The equivalent stiffness of soft boundary for the single-exposure domains can be given by

(S18)

where is the elastic modulus of the surface film in the unexposed domain.

As shown in Fig. S7b, in light of the experimental configurations, the soft boundary conditions of the vertical single-exposure domains can be given by

(S19)

A finite width soft-boundary thin film is bonded to a compliant PDMS substrate with thickness *hs*, where the length, width and thickness of thin film are *L*, *W* and *hf*, respectively and in Fig. S7a. The strains of the surface film can be expressed as

(S20)

where are the strain components in the *x, y* and *xy* directions, respectively.


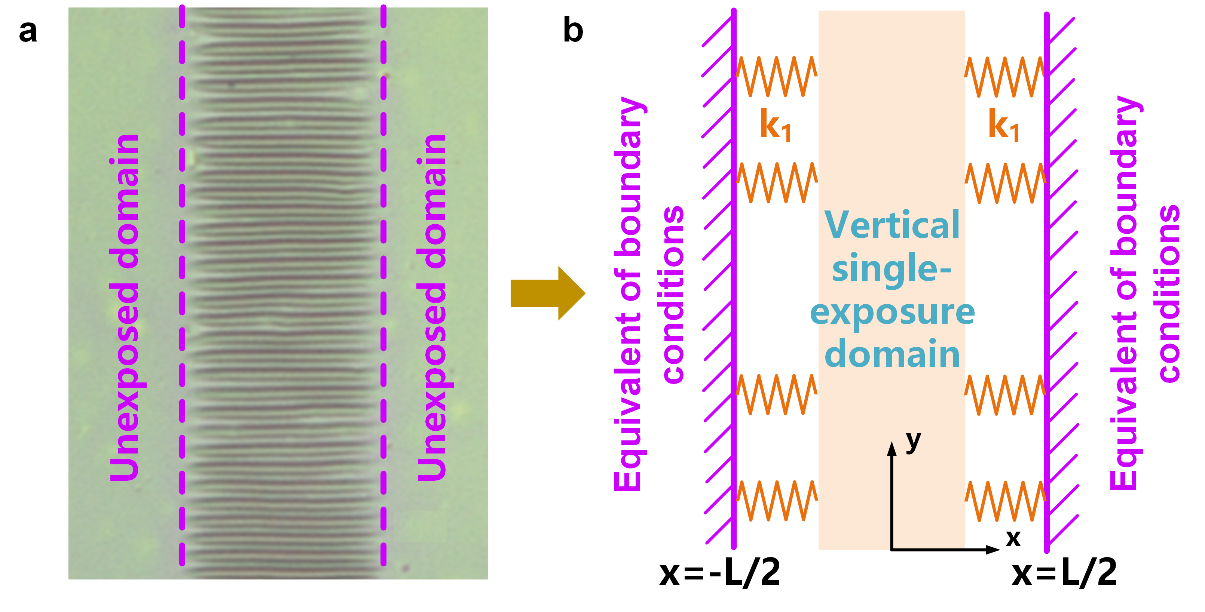


**Figure S7.** Mechanical modelling of soft boundary conditions for the single-exposure domains. (a) the experimental micro image of the single-exposure domains. (b) equivalent mechanical boundaries of the single-exposure domains.

According to the constitutive relation of the top film, the stresses in the thin film can be given by

(S21a)

(S21b)

Due to soft boundaries, thermal-induced residual stress can relax in the *x* direction, which can cause a non-uniform stress distribution along with the width of the film. For one-dimensional relaxation, the stress distribution in the *x* direction can be expressed as

(S22)

where the shear traction at the interface between the film and the substrate , denotes the elastic shear modulus of substrate at the rubbery limit.

Substituting Eq. (21a) into Eq. (22) yields

(S23)

Eq. (S23) is solved with the boundary conditions Eq. (S19), and the stress distributions can be obtained as

(S24a)

(S24b)

where the term of ‘’ is induced by one-direction Poisson’s effect, the term of ‘’ is induced by soft boundary conditions, and the shear-lag length .

**2.4 Principal stress analysis**

The residual thermal stress in the surface film after stress relaxation at the boundary conditions contain three in-plane components and , which can be rewritten by two principle stresses and along with the principal axes *x1* and *x2* (Fig. S8) as follows

(S25)

The corresponding principle angle *θp* in terms of the original stress components can be obtained as [2]

(S26)

**
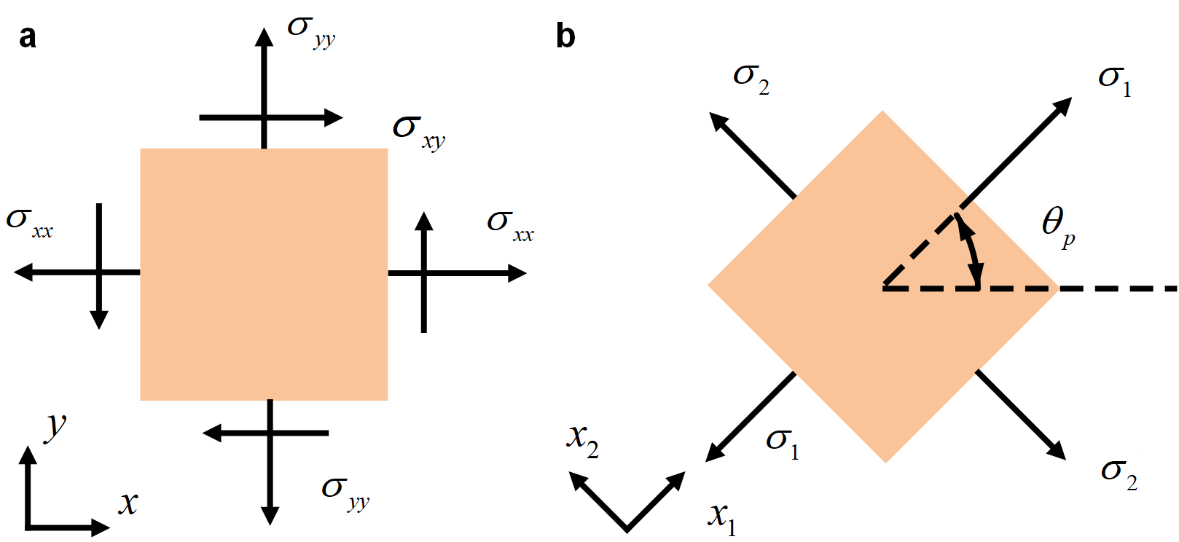
**

**Figure S8.** Residual thermal stress of the surface film represented by a stress element in different orientations: (a) in the *x-y* coordinates, (b)in the principal directions.

**2.5 Equilibrium Wrinkle Patterns**

The out-of-plane displacement of one-dimensional wrinkle pattern along the *y* direction in the domain D2 is given by [3]

(S27)

where the amplitude of wrinkle and the wave numbers . The wave [wavelength](https://www.baidu.com/link?url=MpMXtrcsJu-yEBBwAS1YwOzo3dg5yP0qwqN3eCsbgh5k7ZjZpRFcgWeRuWIuukKYMft3mWe2WaJV2SB90_WERLCETdiJQwxeqACfYfuqECW&wd=&eqid=a77fa65b009d5667000000035d6c6c07) can be expressed asand the critical stress can be obtained as [4].

Because varies along the *x* direction, the amplitude of wrinkle also changes along the *x* direction. For the out-of-plane displacements in D3 and D4, the amplitudes of the wrinkle can be evaluated by replacing the stress ‘’ into the corresponding stresses of the domains. The orientation of the wrinkle can be determined by the direction of the principle stresses.

**2.6 Competitive mechanism analysis**

According to Eq. (S17), all the parameters , , *t* and can be used to tune the stress distributions of surface film, where is the temperature difference of the heat treatment and can control the initial thermal stress via the initial nonlocal physical field; , *t* and are the Poisson’s ratio, exposure time and exposure included angle, respectively. The wrinkle patterns can be determined by stress distributions of the film/substrate system, thus the above four parameters can be employed to regulate the wrinkle morphology.

The peculiar wrinkle pattern evolution phenomenon in Fig. 1b and Fig. 2a-d can be attributed to the competitive mechanism between the three parameters , and *t* when *θe*=90°. As indicated in Eq. (S1), the heating temperature difference can directly impact the initial nonlocal thermal stressin Eq. (S12). Therefore, the heating temperature difference can be used to non-locally control the formation of wrinkle pattern of the whole samples. In this paper, this effect is defined as the thermal stress enhancement effect controlled by the external physical field.

As indicated in Fig. S3, the elastic modulus of surface film (PAN-BA) increases with the exposure time *t*. According to the formula, the critical strain will decrease with the exposure time *t*, which indicates the wrinkles in domain D4 are easier to be triggered. In this paper, this effect is defined as the elastic modulus enhancement effect induced by sequential exposure.

Poisson’s ratio  impacts the formation of wrinkles in the film/substrate system via the bidirectional Poisson's effect. As seen in Eq. (S16), the item ‘’ can be introduced into Eq. (S16) by considering the bidirectional Poisson's effect. Obviously, the stresses in Eq. (S16) can be reduced by ‘’, which can make the stresses in D4 lower than the critical stress , even . In domain D4, both the stresses in Eq. (S16) and the critical stress  can be decreased, thus the wrinkle pattern is regulated as the result of the competitive mechanism between the thermal stress enhancement effect controlled by the external physical field, the elastic modulus enhancement effect by sequential exposures and the bidirectional Poisson's effect.

**3. Experimental results**


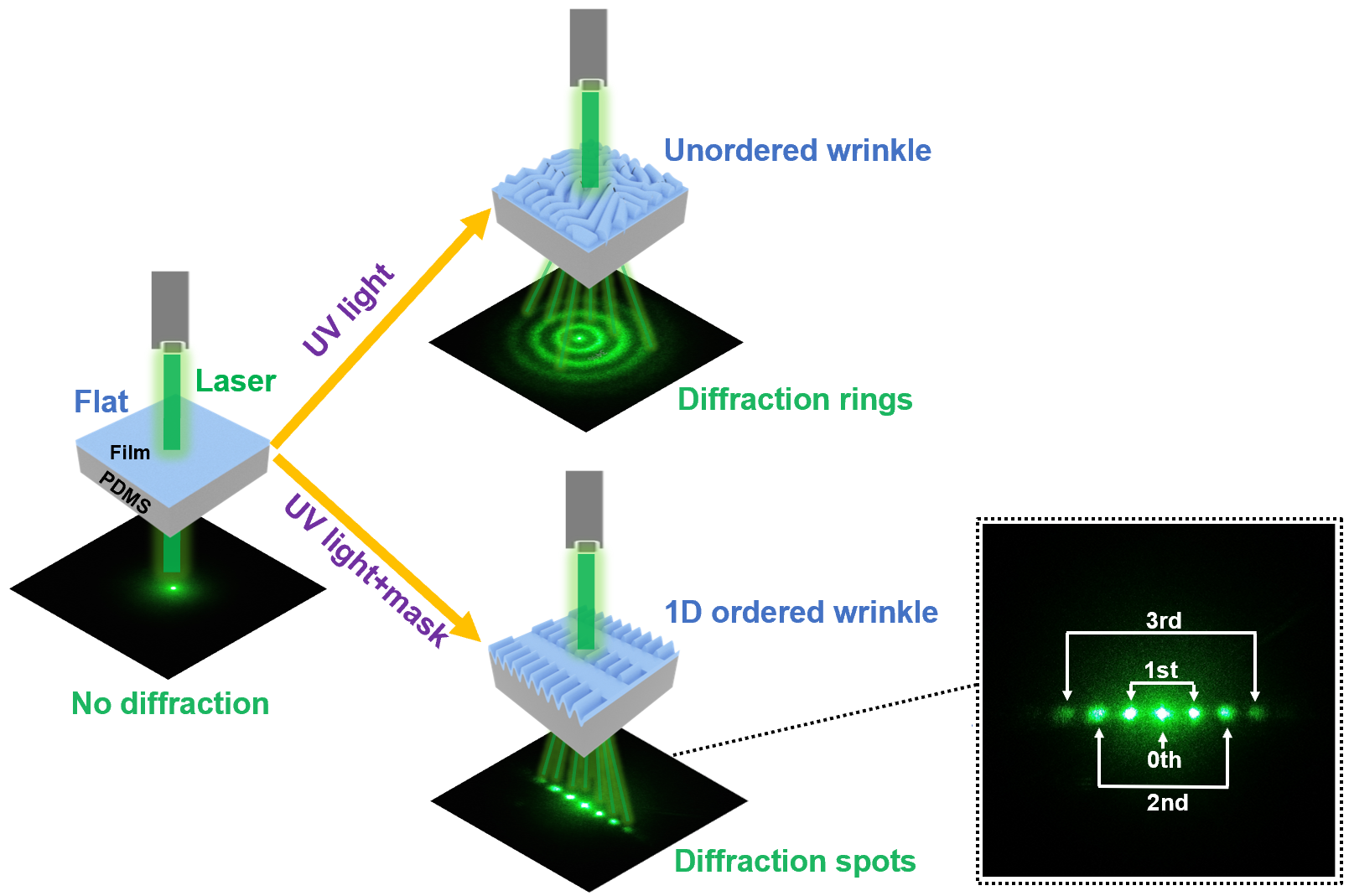


**Figure S9.** Schematic diagram and results of the light diffraction process on the bilayer. The green laser was transmitted through the top film and the PDMS Substrate, then different diffraction patterns could be seen on the black background from the corresponding wrinkle samples.


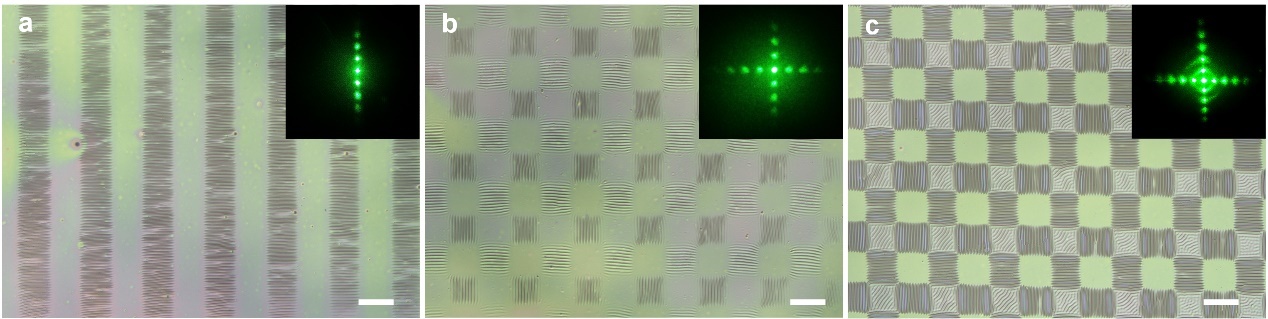


**Figure S10.** The micrographs and diffraction images (the insets) of the wrinkle samples when illuminated by a green laser light. Scale bar=200 µm.


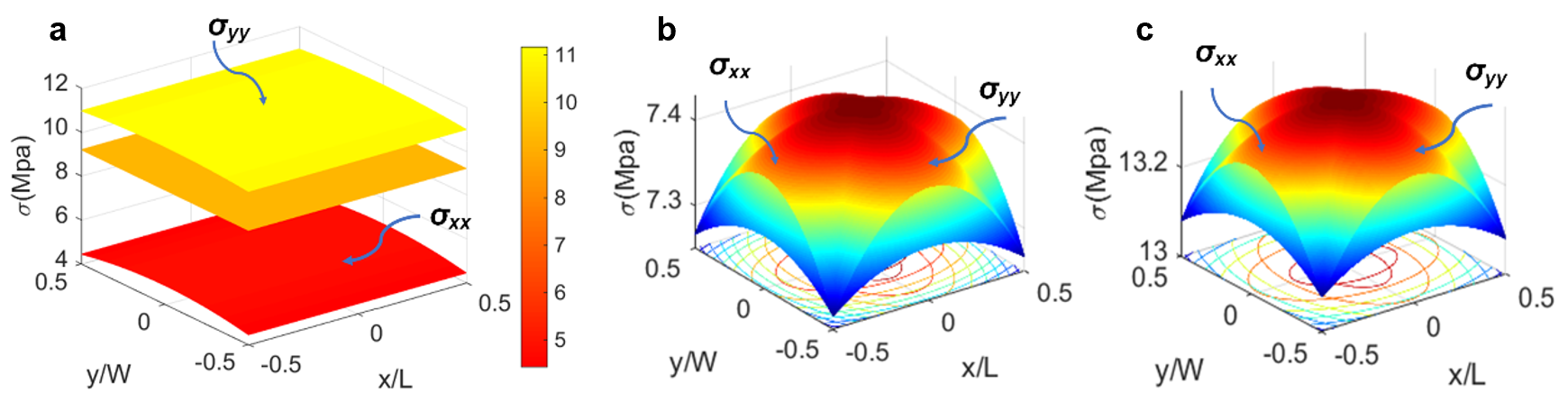


**Figure S11.** The 3D curves of the stress distribution of the wrinkle system with respect of *x* and *y*. (a)The 3D stress distributions of strip wrinkle pattern in the film after first exposure. (b, c)The 3D stress distributions of the film in D4 for two heating temperatures differences °C and °C.


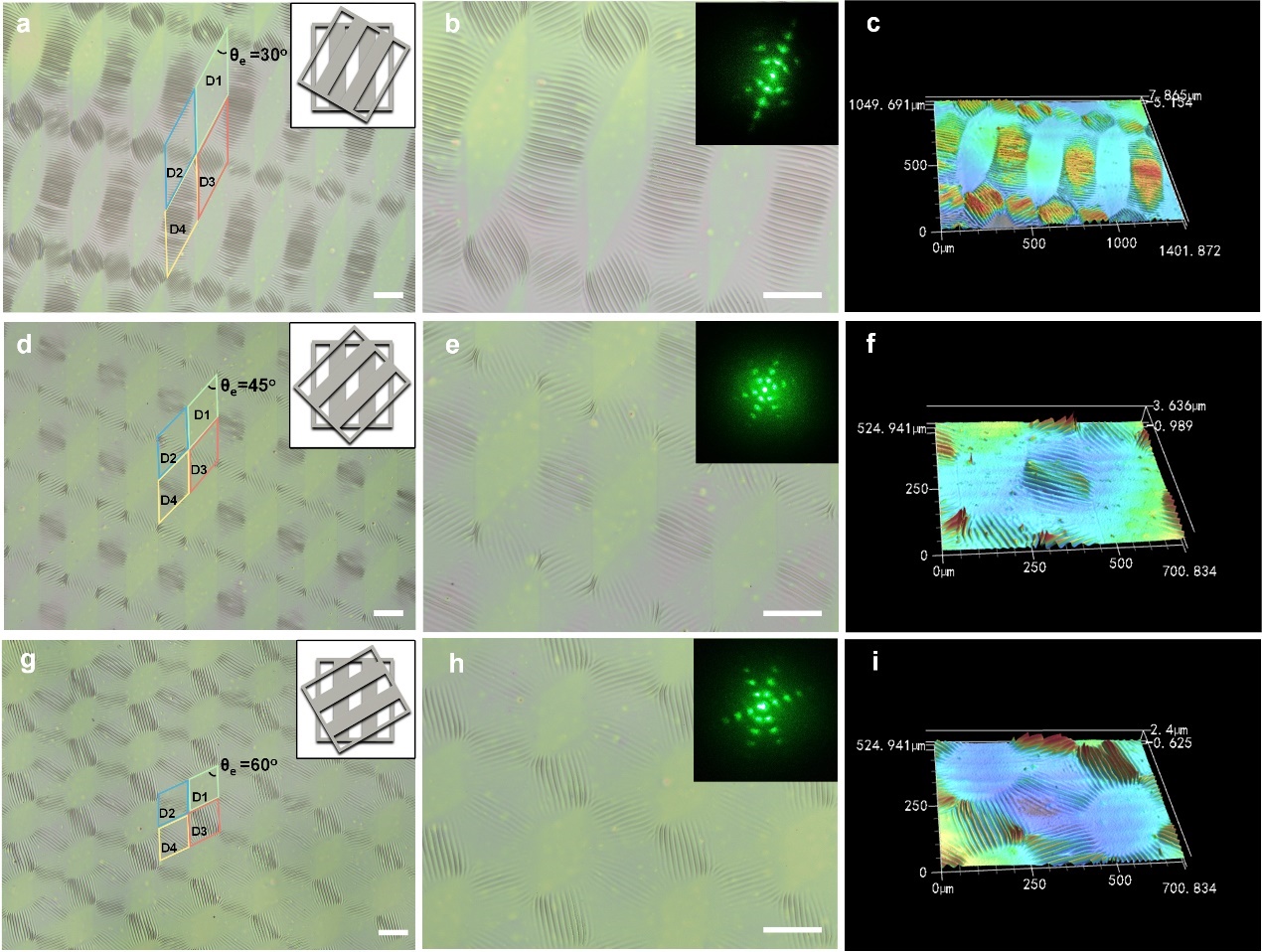


**Figure S12.** Regulation orientation of wrinkles. (a, b, d, e, g, h) 2D micrographs of the wrinkles fabricated with *θe = 30°*, *45°*, *60°*, respectively. The insets are the twice laying directions of photomask and the corresponding diffraction patterns. Scale bars=200 µm. (c, f, i) The corresponding 3D LSCM images of wrinkles.


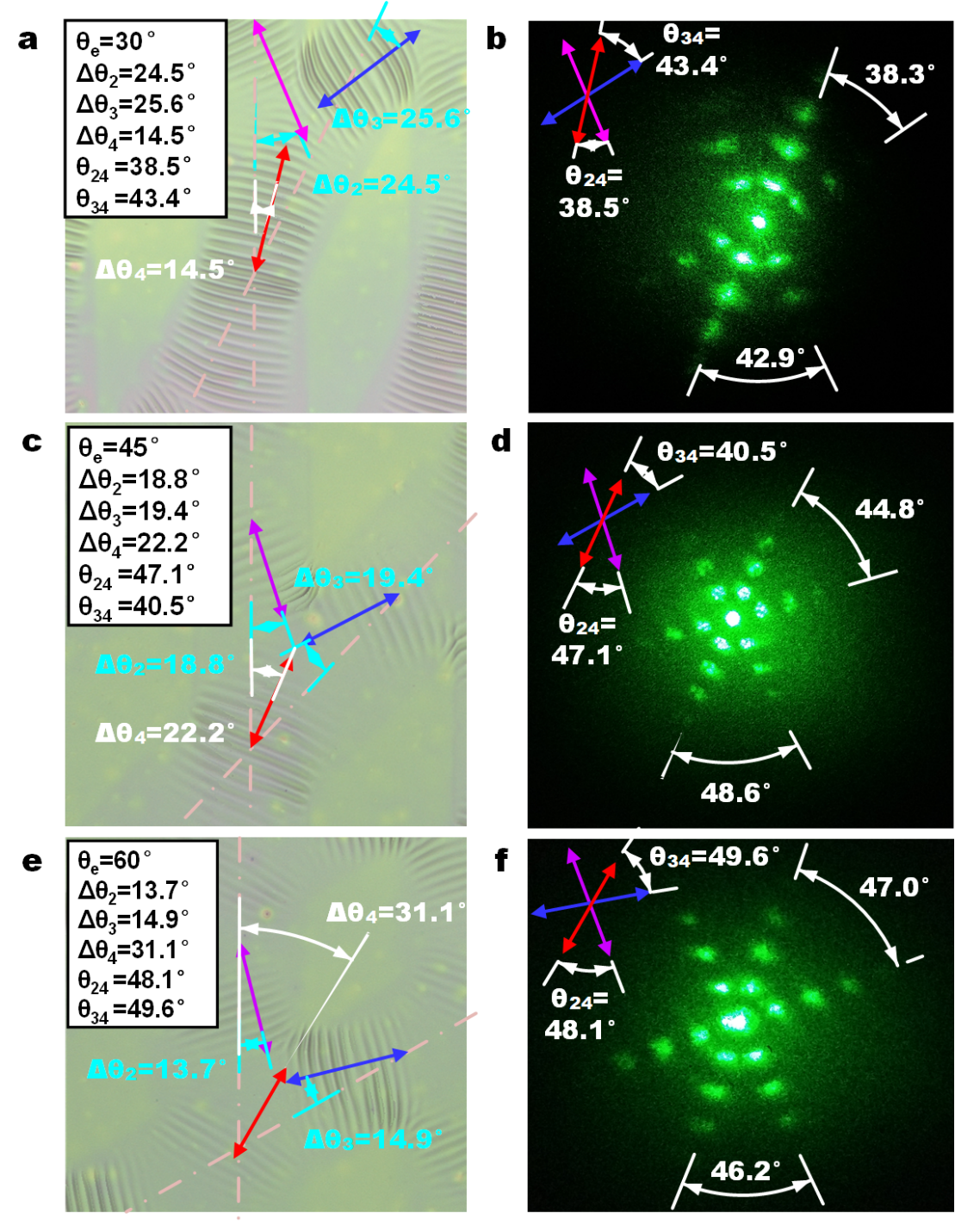


**Figure S13.** The included angles of wrinkle orientations in different exposure domains for different *θe* (=30°, 45°, 60°).


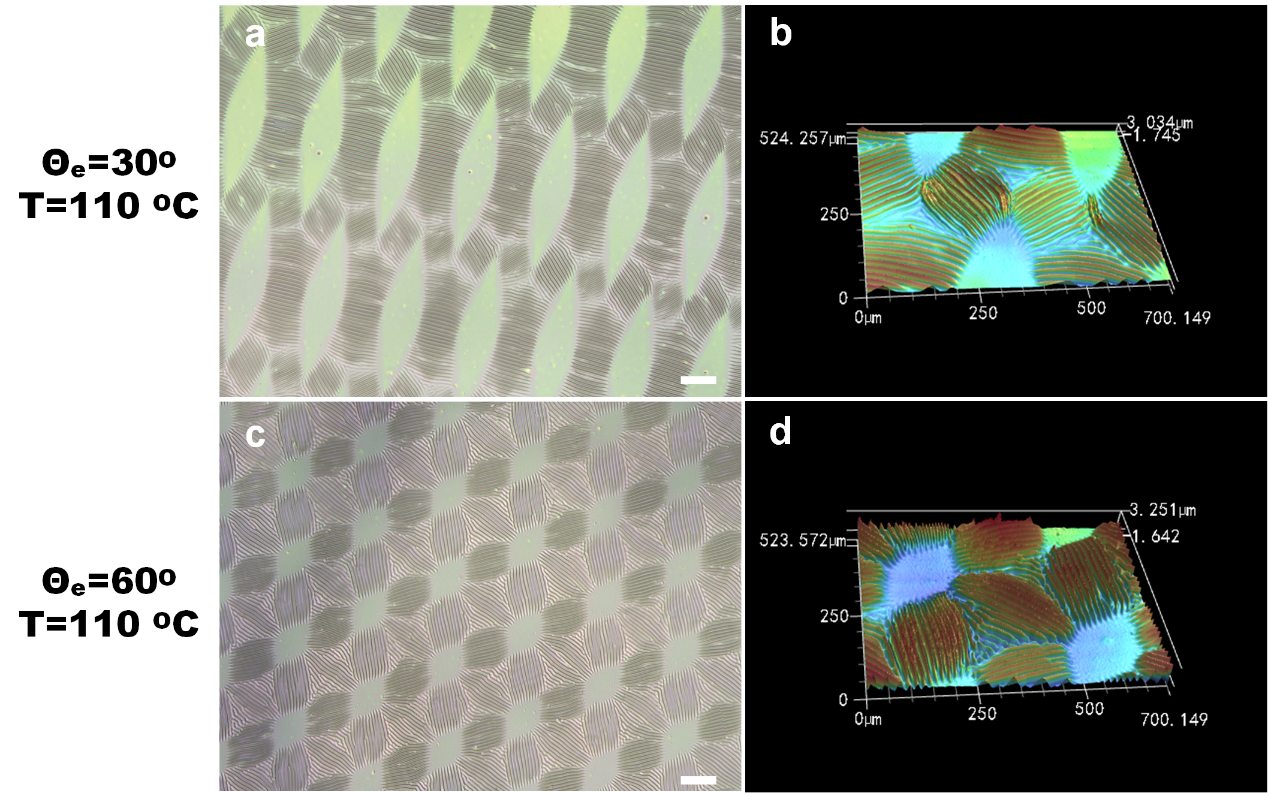


**Figure S14.** The 2D micrographs and 3D LSCM images of the wrinkle samples. Scale bar=200 µm.


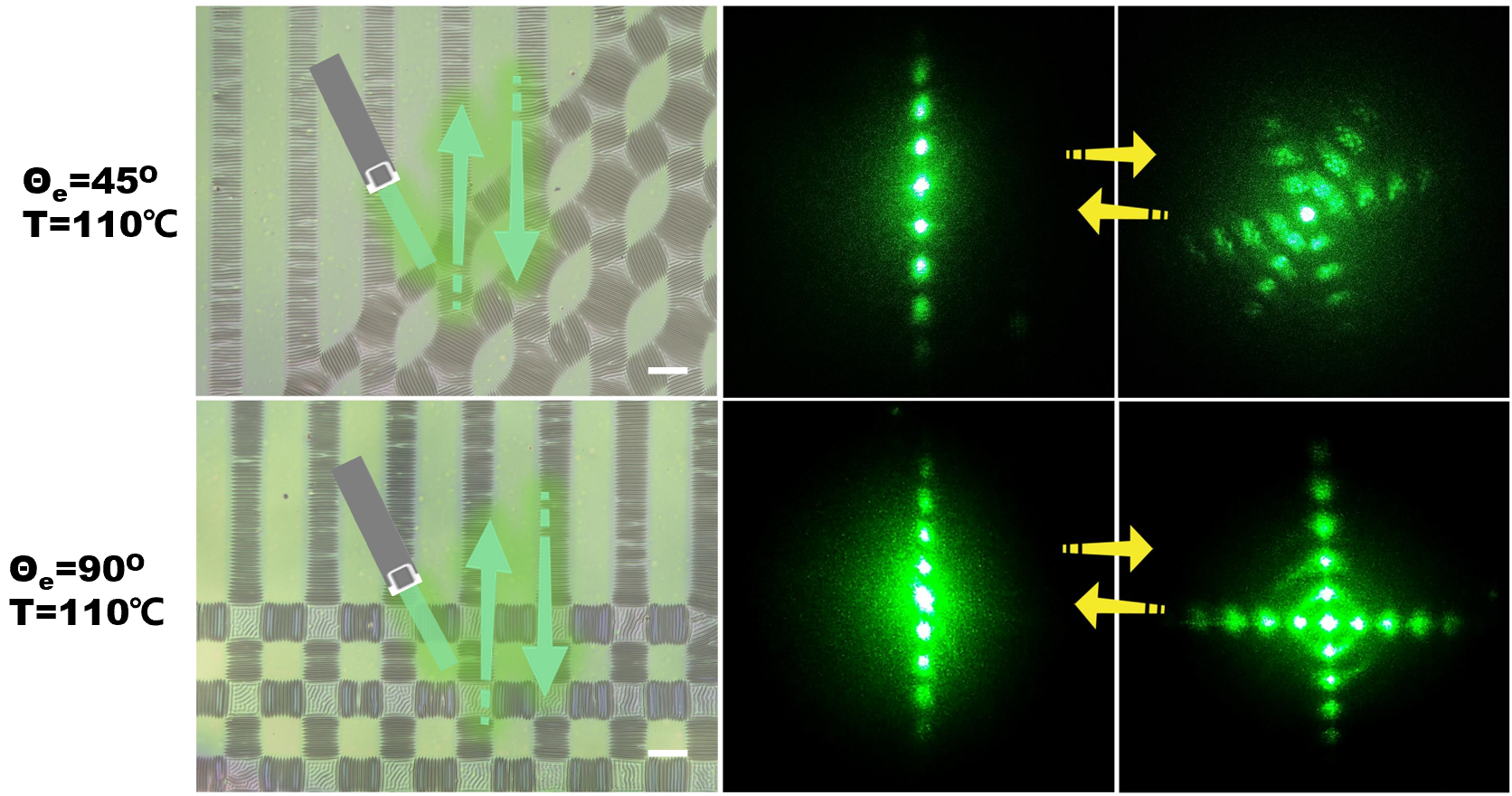


**Figure S15.** The dynamic transition of diffraction images on the wrinkle samples when the green laser light moved back and forth over different domains of wrinkle surface. Scale bar=200 µm.


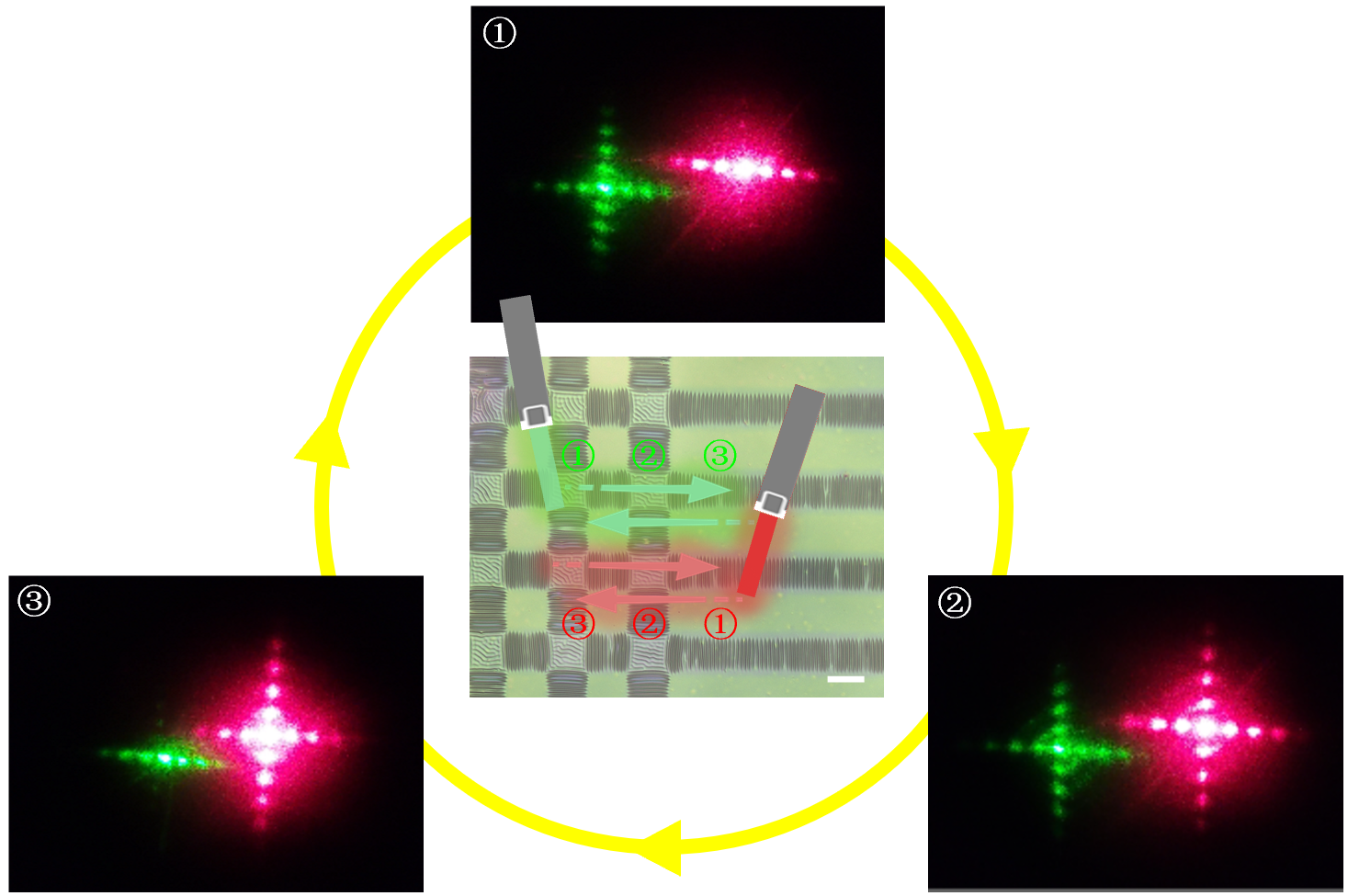


**Figure S16.** The dynamic transition of diffraction images on the wrinkle sample when a green laser light and a red laser light moved back and forth over different domains of wrinkle surface. Scale bar=200 µm.


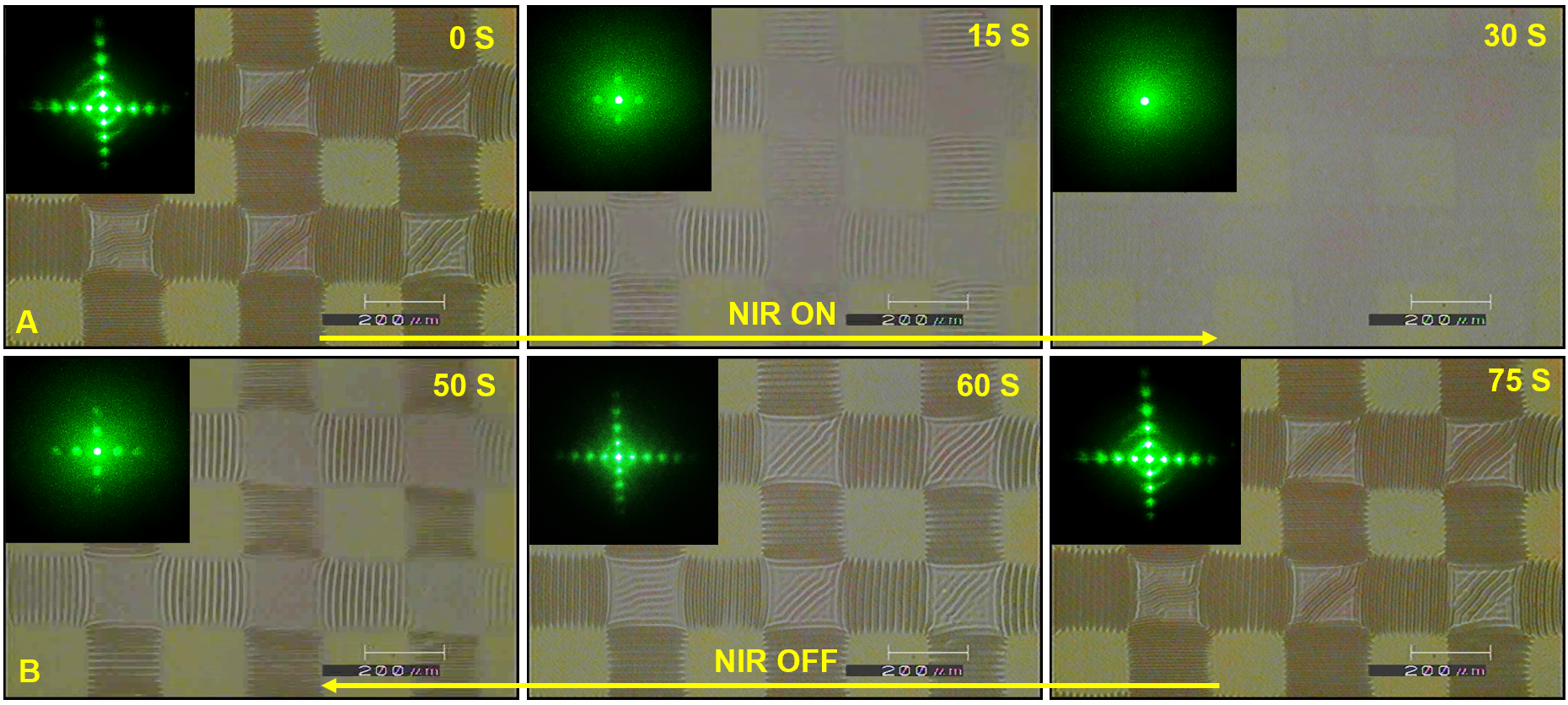


**Figure S17.** Dynamics of wrinkle extinction/formation process via NIR switchable on/off cycle. (A) Optical images of wrinkle pattern and the corresponding diffraction pattern disappearing behavior via NIR irradiation. (B)Optical images of recovery evolution process after removing of NIR. The NIR intensity was 1.5 W/cm2. Scale bar=200 µm.


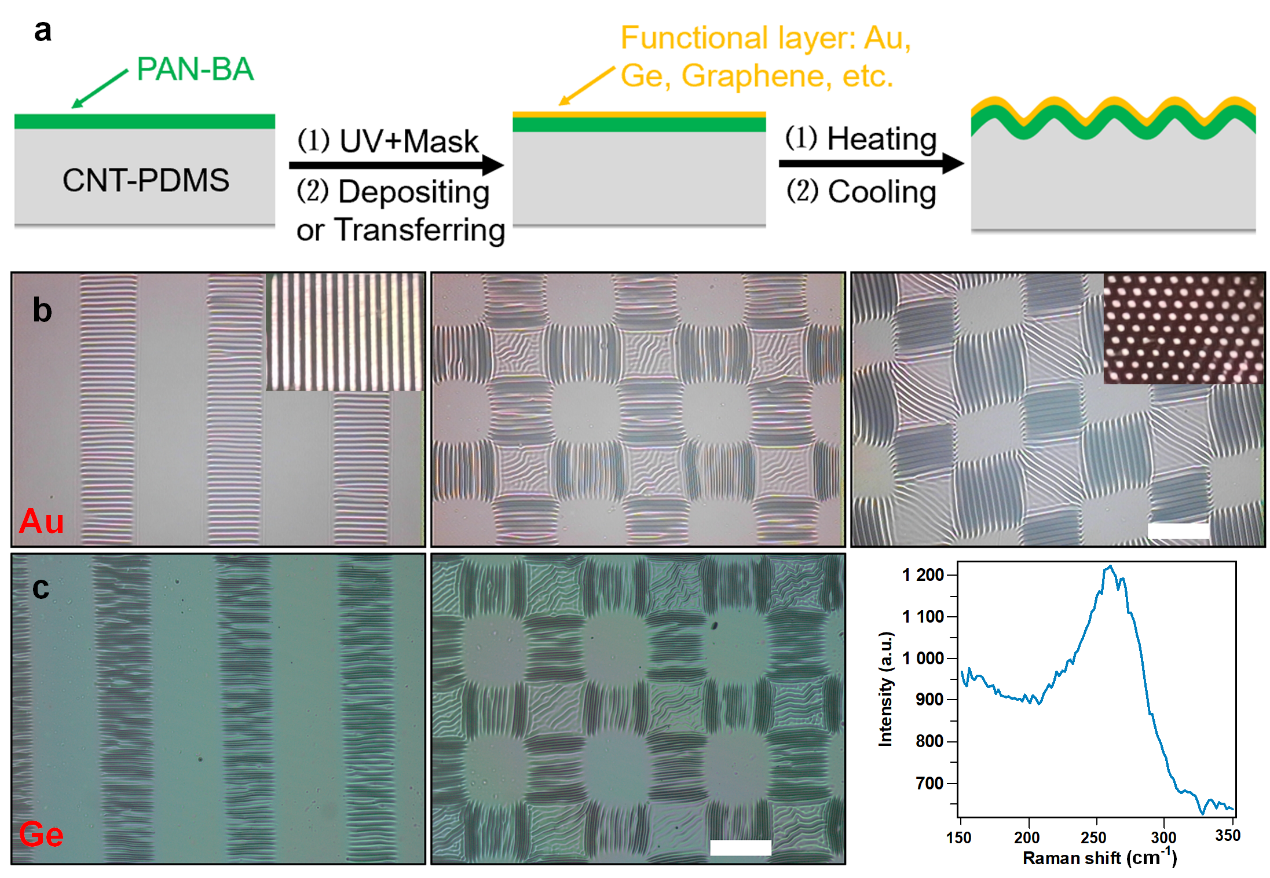


**Figure S18.** NIR-driven dynamic template.(a) Schematic illustration the fabrication process of NIR-driven dynamic three-layer wrinkled platform. (b) Various of Au-functionalized ordered wrinkles. The insets are the corresponding macrophotographs by camera. (c) Various of Ge-functionalized ordered wrinkles. Where the existence of Raman characteristic peak (~280 cm-1) indicates that Ge had been deposited onto the surface of wrinkled bilayer system.

**Movies**

Movie S1. The dynamic transformation of light diffraction patterns on different wrinkled micro-domains (Single green light).

Movie S2. The dynamic transformation of light diffraction patterns on different wrinkled micro-domains (Green and red light).

Movie S3. Wrinkle pattern’s (*θe=*45°*,* *T=*110 °C) elimination/regeneration cycles in microscope.

Movie S4. Wrinkle pattern’s (*θe=*90°*,* *T=*110 °C) elimination/regeneration cycles in microscope.

Movie S5. The evolution of wrinkle’s (*θe=*45°*,* *T=*110 °C) light diffraction patterns upon the NIR on/off switch.

**References**

1. Bowden N, Brittain S and Evans AG *et al.* Spontaneous formation of ordered structures in thin films of metals supported on an elastomeric polymer. Nature 1998; **393**: 146-149.

2. Im SH & Huang R. Wrinkle patterns of anisotropic crystal films on viscoelastic substrates. *J. Mech. Phys. Solids* 2008; **56**: 3315-3330.

3. Song J, Jiang H and Choi WM *et al.* An analytical study of two-dimensional buckling of thin films on compliant substrates. *J. Appl. Phys.* 2008;**103**: 014303.

4. Huang Z, Hong W and Suo Z. Nonlinear analyses of wrinkles in a film bonded to a compliant substrate. *J. Mech. Phys. Solids* 2005; **53**: 2101-2118.
